# Is risk-stratified breast cancer screening economically efficient in Germany?

**DOI:** 10.1371/journal.pone.0217213

**Published:** 2019-05-23

**Authors:** Matthias Arnold, Katharina Pfeifer, Anne S. Quante

**Affiliations:** 1 Centre for Health Economics, University of York, York, United Kingdom; 2 Munich Center of Health Sciences, Ludwig-Maximilians-Universität, Munich, Germany; 3 Institute of Health Economics and Health Care Management, Helmholtz Zentrum München – German Research Center for Environmental Health, Neuherberg, Germany; 4 Frauenklinik, Klinikum Rechts der Isar, Technical University Munich (TUM), Munich, Germany; University of Bern, SWITZERLAND

## Abstract

**Objectives:**

Risk stratification has so far been evaluated under the assumption that women fully adhere to screening recommendations. However, the participation in German cancer screening programs remains low at 54%. The question arises whether risk-stratified screening is economically efficient under the assumption that adherence is not perfect.

**Method:**

We have adapted a micro-simulation Markov model to the German context. Annual, biennial, and triennial routine screening are compared with five risk-adapted strategies using thresholds of relative risk to stratify screening frequencies. We used three outcome variables (mortality reduction, quality-adjusted life years, and false-positive results) under the assumption of full adherence vs. an adherence rate of 54%. Strategies are evaluated using efficiency frontiers and probabilistic sensitivity analysis (PSA).

**Results:**

The reduced adherence rate affects both performance and cost; incremental cost-effectiveness ratios remain constant. The results of PSA show that risk-stratified screening strategies are more efficient than biennial routine screening under certain conditions. At any willingness-to-pay (WTP), there is a risk-stratified alternative with a higher likelihood of being the best choice. However, without explicit decision criteria and WTP, risk-stratified screening is not more efficient than biennial routine screening. Potential improvements in the adherence rates have significant health gains and budgetary implications.

**Conclusion:**

If the participation rate for mammographic screening is as low as in Germany, stratified screening is not clearly more efficient than routine screening but dependent on the WTP. A more promising design for future stratified strategies is the combination of risk stratification mechanisms with interventions to improve the low adherence in selected high-risk groups.

## Introduction

When evaluating national mammography screening programs, it remains controversial whether the benefits outperform the risks, such as false-positive screening results and unnecessary diagnostic procedures [1–4]. As trade-offs between risks and benefits may vary at the individual level, an individualized program is very likely to represent a more effective approach than population-wide criteria for screening. On one hand, it is desirable to avoid unnecessary diagnostic procedures involving radiation in women who are unlikely to develop breast cancer. On the other hand, there is a need to intensify diagnostic procedures for women at higher risk. It is desirable to develop risk-stratified screening intervals based on the individual risk profile, taking into account family history, breast density, and breast biopsies [5–8].

Recent studies have taken up the challenge of evaluating risk-stratified screening proposals [5–10]. However, all evaluations were made under the assumption of full adherence [5–10]. In our previous work [11], we were able demonstrate that the performance of screening programs is sensitive to changes in the adherence assumption. Thus, assuming full adherence biases the simulation results.

In Germany, women between 50 and 69 years are routinely invited to participate in mammography screening every two years. The participation rate in the mammography screening program is especially low at 54% [12] and does not reach the quality assurance goal of 70% set within the European guidelines [13]. Therefore, it is essential to consider the actual participation rate in an economic evaluation of risk-adapted screening strategies vs. routine screening.

This economic evaluation evaluates both routine (with annual, biennial, or triennial intervals) and proposals for stratified screening strategies (based on a narrow selection of risk factors found in the general population). We use a simulated German population in a Markov decision model to compare these screening strategies under the assumption of full adherence vs. an adherence rate of 54%.

## Methods

We adapt a micro-simulation Markov model to represent the population and health system in Germany. The model randomly selects different health states for women who are initially healthy. Over the course of a lifetime, women may develop ductal carcinoma in situ (DCIS), invasive breast cancer, or remain healthy and die from other causes. Women with DCIS may or may not develop invasive cancer later on. Women with breast cancer may die from disease or other causes. The probability of developing invasive breast cancer increases with age, high density of the breast tissue (at 50 years of age, 48.3% of women have heterogeneously dense and 6.9% have extremely dense tissue) [14], a positive history of breast cancer in a first-degree relative (16.1% of women) [15], or previous biopsies (28.2% of women) [15]. Women can be diagnosed with breast cancer in one of three stages: local, regional, or distant. The stage distribution at diagnosis depends on the frequency with which mammography screening was performed, age and breast density. The stage at time of diagnosis influences the annual survival probability. Simulations run from a start age of 50 years (the age of the first screening invitation for women in Germany [16]) until the end of life or 100 years for a total of 3,000,000 women, which produces stable results comparing within-strategy variance and between-strategy variance [17]. An overview of the parameters used is given in Table 1 and described in detail in the following. Additional descriptions regarding the adaptation from the original model and the state transition diagram can be found in section *Model description* in the S1 Appendix.

**Table 1:** Model input parameters.

Parameter	Description	Source
**Population demographics**		
Background mortality	Mortality in absence of breast cancer. Based on life tables and cause of death statistics.	[20]
**Natural history of breast cancer**		
Incidence of breast cancer in absence of screening	Based on APC models to correct for age, period, and cohort effects in national cancer registry data.	[18]
Stage distribution	Stage distribution from detected cancer cases by age and screening interval. Based on calculations from BCSC data.	[5, 15, 34]
Survival time	Based on survival curves from Munich Tumor Registry data.	[23]
**Breast cancer screening**		
Mammography adherence	Assumed full adherence and reported participation rates in the Mammography Evaluation reports.	[16]
Sensitivity of mammography	Based on age and breast density. Mammographic vs. clinically detected cancers are expressed in the stage distributions.	[5, 15, 34]
Specificity of mammography	False-positive mammograms are calculated based on data from the Mammography Evaluation reports.	[16]
Prevalence of breast density	Based on the literature.	[14, 22]
Risk levels for risk factors	Relative risks of breast density, family history in first-degree relative, and previous biopsies are based on the literature and BCSC risk calculator.	[5, 15, 35]
Cost of screening and diagnostic work-up	Based on the price catalog for ambulatory care (EBM).	[36]
Health utility effects of screening and diagnostic work-up	Based on EQ-5D tariff-based utility scores for mammography screening, core needle biopsy, and vacuum biopsy. Biopsy is weighted using probability of core needle and vacuum biopsy reported in the Mammography Evaluation reports.	[16, 31, 37]
**Breast cancer treatment**		
Treatment use	Based on treatment pathways for triple negative, hormone receptor-positive, and estrogen receptor-positive women reported in an analysis from the German Consortium for Hereditary Breast and Ovarian Cancer. Adapted for women of moderate risk using guidelines, disease management programme evaluation reports, and the literature.	[24, 25, 38–41]
Treatment effect	Assumed to be included in the stage-dependent survival times.	[23]
Cost of treatment	Based on estimates based on an analysis from the German Consortium for Hereditary Breast and Ovarian Cancer using the price catalog for stationary care in a German cohort. Adapted for women of moderate risk using guidelines and the literature.	[24, 42, 43]
Health utility effect of breast cancer and treatment	Based on EQ-5D tariff-based estimates reported in the literature.	[5, 27]

### Incidence, survival, and mortality

We estimate invasive breast cancer and DCIS incidence from the German Center for Cancer Registry Data (ZfKD) [18] and population estimates from the Federal Statistical Office using age–period–cohort models (APC) [19], which allow calculation of age-specific incidence rates while controlling for age, period, or cohort effects [20]. Details can be found in the sections *Invasive cancer incidence* and *DCIS incidence* of the S1 Appendix. Breast cancer incidence is modified by relative risk from three risk factors: family history of breast cancer [5, 21], personal history with biopsy [5, 21], and breast density [5, 15]. Relative risks are calculated as the risk in developing breast cancer in the group carrying the risk factor over the risk of developing breast cancer in the risk not carrying the risk factors. These risk factors are chosen as readily available representatives which are found in the general population. Each woman is randomly assigned a risk combination at the start of the simulation. Breast density may change every 5 years [22], which reflects empirically observed changes in breast tissue density [14]. Section *Risk factors* in the S1 Appendix describes the prevalence of risk factors in the population, the changes in breast density, and the associated relative risks.

Survival in our simulation is dependent on the “Markov stage”: for healthy women or women with DCIS, survival is calculated from the age-specific mortality rate from causes other than breast cancer using data from the Federal Statistical Office [20]. The exact calculation is described in section *Background mortality* in the S1 Appendix. For women with invasive breast cancer at stages of local, regional, or distant cancers, mortality rates are determined by stage-specific mortality rates. For each stage, we accordingly describe the cancer-specific survival or mortality rates using data from the Munich Tumor Registry [23]. Details can be found in section *Breast cancer mortality* in the S1 Appendix.

### Cost and utility parameters

We chose the payer perspective of the statutory health insurances. All prices are standardized to 2017 Euros, and the discount rate are set to 3% for cost and utility parameters. Prices are based on the national price catalog (EBM), diagnosis-related groups (DRG), or published literature. For the mammography screening and associated additional procedures, we calculate the costs as follows: mammography screening (€73.50), additional follow-up (€94.45), core needle (€21.10), or vacuum biopsy (€39.79), section *Diagnostic work up* in the S1 Appendix describes the probability used for each technology. Details of the EBM codes and prices can be found in section *Cost parameters* of the S1 Appendix. Treatment pathways are based on estimates by Muller, Danner [24] for German breast cancer (BRCA) susceptibility gene carriers and adapted their treatment probabilities to reflect women over 50 years without BRCA gene mutation. We use a classification of molecular subtypes, which are associated with distinct treatment pathways in local, regional, or distant cancers. Based on Liedtke, Rody [25], we distinguish between triple negative (HER2/neu negative, estrogen receptor negative, and progesterone negative), hormone receptor negative and HER2/neu positive, and hormone receptor positive with or without HER2/neu positive. We include surgical treatment (either mastectomy or breast-conserving therapy), chemotherapy (plus Trastuzumab for HER2/neu), chemotherapy-induced adverse events, endocrine therapy, and radiotherapy after surgery and palliative care. All hospital-based costs are based on German DRG aggregates. Probabilities, literature sources, and sensitivity ranges can be found in section *Cost parameters* of the S1 Appendix.

For health utilities, we use EQ-5D estimates based on the time-trade-off tariff of Dolan [26]. Utility parameters for healthy women and breast cancer patients are based on EQ-5D estimates from Lidgren, Wilking [27]. Losses in quality-adjusted life years (QALY) for screening and diagnostic work up are based on literature. Section *Utility parameters* in the S1 Appendix describes the literature used, the exact calculation, and the sensitivity ranges.

### Screening strategies and adherence

Our risk stratification approach is to simulate screening intervals of 1 year, 2 years, or 3 years. We use breast density-specific stage distribution for these intervals from the Breast Cancer Surveillance Consortium (BCSC) [15]. We evaluate three routine screening strategies for annual, biennial, and triennial intervals and a total of five risk-stratified strategies, where the interval is based on the risk profile. These screening strategies were designed to represent threshold based strategies with a degree of variation in the total number of screening events. This variation reflects different motives in decision makers (representing reduction or increase in screening efforts on average). Based on the literature, we chose four different thresholds of relative risks (RR) of 2, 1.5, 1, and 0.5 [28, 29] to create five combinations of high-, medium-, and low-risk groups:

- Strategy RR 2–1 suggests annual screening for women with relative risk above 2.0, biennial screening for women with relative risk between 1.0 and 2.0, and triennial screening for women with relative risk below 1.0.
- Strategy RR 1–0.5 suggests annual screening for women with relative risk above 1.0, biennial for women with relative risk between 0.5 and 1, and triennial for women with relative risk below 0.5.
- Strategy RR 1.5–1 suggests annual screening for women with relative risk above 1.5, biennial for women with relative risk between 1.5 and 1.0, and triennial for women with relative risk below 1.0.
- Strategy RR 1.5–0.5 suggests annual screening for women with relative risk above 1.5, biennial for women between 0.5 and 1.5, and a lower threshold of 0.5 for triennial screening.
- Strategy RR 2–0.5 suggests annual screening for women with a relative risk above 2.0, biennial screening for women between 2.0 and 0.5, and triennial screening for women below 0.5.

Section *Screening strategies* in the S1 Appendix illustrates how these clusters transfer to risk factor and age combinations. These five strategies are compared under two adherence assumptions: full adherence and the actual adherence rate in Germany of 54% [16]. Under the assumption of the adherence rate of 54%, whether a woman attends screening or not is randomly distributed. If a woman is diagnosed with breast cancer and has participated in screening, then her cancer stage is assigned based on the stage distribution of the respective screening interval. If a woman has been diagnosed with breast cancer and has not participated in screening, then her cancer stage is assigned based on the same stage distribution as women who do not attend screening at all. *Section Mammography screening and adherence* in the S1 Appendix discusses the technical implementation.

### Benefits and harms

We calculate incremental cost and incremental effect measures for three outcomes: breast cancer mortality reduction, QALY, and false-positive screening results. As a representation of false-positive screening results, we tracked the number of biopsies only necessary to clarify false-positive screening results, as they significantly affect quality of life [30–33]. Overdiagnosis was implemented as harmful screening effect relative to the number of the screening events.

### Uncertainty and probabilistic sensitivity analysis

We use univariate and probabilistic sensitivity analyses (PSA) to test parameter uncertainty on the model robustness. The complete list of variables and the sensitivity ranges used are described in section *Distribution and ranges for sensitivity analysis* of the S1 Appendix. For the PSA, 500 runs with 3,000,000 trials were used, which were found to produce stable variance with manageable computation time (of roughly 800 hours for the PSA with TreeAge Pro 2017 on an Intel i5 computer cluster with 32 cores). With cost-effectiveness acceptability curves (CEAC), we compare PSA results over multiple outcomes as suggested by Stollenwerk, Lhachimi [44].

### Efficiency criteria

As suggested by the Institute for Quality and Efficiency in Health Care (IQWIG) [45], we do not use a specific willingness-to-pay (WTP) threshold, but assess whether the strategies are technically efficient alternatives compared with existing strategies. The efficiency frontier describes the best combination of incremental cost and effect [46]. Efficient strategy would be placed on the efficiency frontier, whereas inefficient proposals would be below the frontier line.

For the PSA, we assess whether the CEAC lies strictly above the alternative strategy. In this situation, the probability of being cost-effective for the first strategy is consistently higher than its alternative. It would then be efficient to decide on the first strategy. In situations where the CEACs intersect, the strategies change their rank if a certain WTP threshold is crossed. A clearly defined WTP threshold is then necessary for the decision between two alternatives. In the absence of such a clearly defined WTP, none of the alternatives is clearly efficient. We calculate the expected value of perfect information (EVPI) to identify the monetary value of uncertainty in the model.

## Results

**Fig 1:**
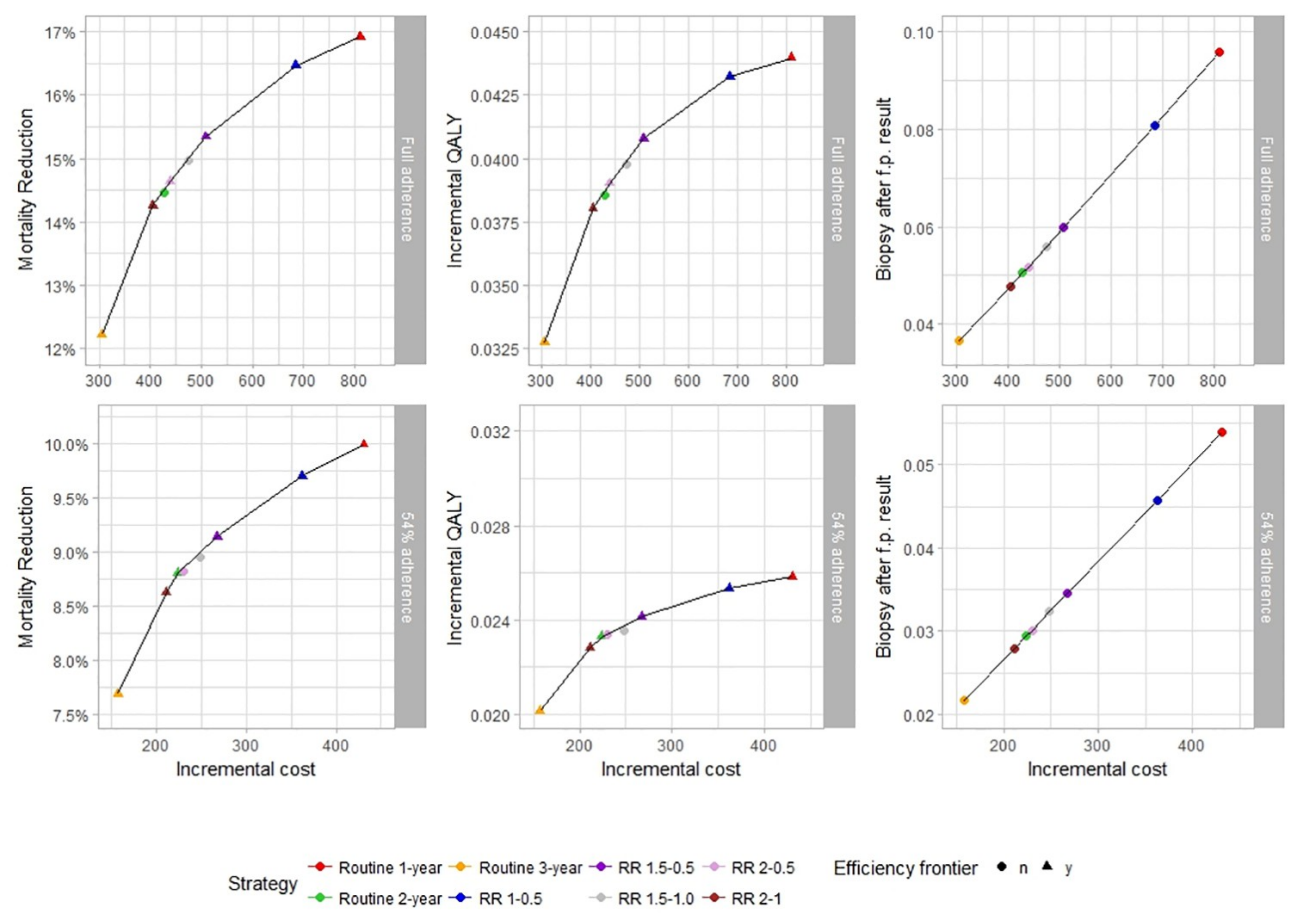
Cost-effectiveness efficiency frontiers.

Table 2 presents the outcomes and costs of each screening strategy as increments compared with a situation without screening under the assumption of full adherence vs. 54% adherence. Fig 1 shows the efficiency frontiers representing outcomes for each strategy under the assumption of full adherence vs. 54% adherence. The axis were standardized between the adherence assumptions to represent the same ratio. The cost in Fig 1 represents additional spending for each screening strategy. Each strategy produces mortality reduction and QALY, but also false-positive screening results, which leads to unnecessary recall screening and in some cases unnecessary biopsies.

**Table 2:** Outcomes and costs as increments per strategy vs. no screening, mean (confidence interval) per woman.

Adherence	Strategies	Mortality reduction (in %)	Incremental QALY	Biopsies after false-positive screening	Incremental cost (2017 Euro)
Full Adherence	Routine 3-year	12.22 (12.26–12.19)	0.033 (0.033,0.033)	0.037 (0.036,0.037)	305.18 (305.15,305.22)
	Routine 2-year	14.46 (14.5–14.42)	0.039 (0.039,0.039)	0.050 (0.050,0.051)	427.8 (427.76,427.83)
	Routine 1-year	16.92 (16.96–16.87)	0.044 (0.044,0.044)	0.096 (0.095,0.096)	810.84 (810.85,810.83)
	RR 2–1	14.26 (14.3–14.22)	0.038 (0.038,0.038)	0.048 (0.048,0.048)	404.48 (404.44,404.53)
	RR 1–0.5	16.46 (16.51,16.42)	0.043 (0.043,0.043)	0.081 (0.080,0.081)	684.76 (684.75,684.78)
	RR 2–05	14.64 (14.68,14.6)	0.039 (0.039,0.039)	0.052 (0.051,0.052)	438.52 (438.48,438.56)
	RR 1.5–1.0	14.96 (15,14.92)	0.040 (0.040,0.040)	0.056 (0.056,0.056)	474.15 (474.11,474.2)
	RR 1.5–0.5	15.34 (15.39,15.3)	0.041 (0.041,0.041)	0.060 (0.060,0.060)	508.42 (508.38,508.46)
54% Adherence	Routine 3-year	7.68 (7.71,7.66)	0.020 (0.020,0.020)	0.022 (0.022,0.022)	157.14 (157.12,157.16)
	Routine 2-year	8.81 (8.83,8.78)	0.023 (0.023,0.023)	0.030 (0.029,0.030)	224.12 (224.1,224.14)
	Routine 1-year	9.99 (10.02,9.97)	0.026 (0.026,0.026)	0.054 (0.054,0.054)	430.75 (430.75,430.74)
	RR 2–1	8.63 (8.66,8.61)	0.023 (0.023,0.023)	0.028 (0.028,0.028)	211.13 (211.1,211.15)
	RR 1–0.5	9.7 (9.73,9.68)	0.025 (0.025,0.025)	0.046 (0.045,0.046)	362.46 (362.45,362.47)
	RR 2–05	8.82 (8.85,8.8)	0.023 (0.023,0.023)	0.030 (0.030,0.030)	229.97 (229.95,229.99)
	RR 1.5–1.0	8.95 (8.98,8.93)	0.024 (0.024,0.024)	0.032 (0.032,0.033)	248.55 (248.53,248.58)
	RR 1.5–0.5	9.14 (9.17,9.12)	0.024 (0.024,0.024)	0.035 (0.034,0.035)	267.54 (267.51,267.56)

Under the assumption of full adherence, routine biennial screening produces 14.46% mortality reduction and 0.0386 incremental QALY (equivalent to 14 days in perfect health) at a cost of €427.8 per woman (Table 2). If adherence is 54%, routine biennial screening reduces mortality by 8.81% and increases quality of life by 0.0233 at a cost of €224.12. Mortality reduction is thus reduced by 39%, QALYs by 40%, and cost by 48%. When comparing this with the other strategies, we find that the relative impact of non-adherence on performance and cost is very homogeneous with only small variations. Adherence consistently affects performance and cost, which is why incremental cost-effectiveness ratios (ICERs) remain consistent.

Overall, the performance of the screening strategies is reduced by approximately 40% but, at the same time, about 48% costs are saved. This explains why the slope of the QALY efficiency frontier (Fig 1) is less steep with 54% adherence than with full adherence. Thus, the investment in more intensive screening differs under the assumption of full vs. 54% adherence. As an example: changing from triennial routine screening to annual screening costs additional €505.66 and returns additional 0.0116 QALYs if full adherence is assumed, which is a ratio €43,591 per QALY. If adherence is 54% though, the same investment costs €273.61 for 0.0057 QALY, which is a ratio of €48,001 per QALY. Adherence thus affects the returns on the screening investment.

The subgroup analysis in Table 3 shows the performance of the risk strategies within the risk groups. Small variations between risk groups in different strategies but with identical screening recommendations are to be expected due to the randomization of the micro-simulation (for example the difference in results between the annual risk group in RR2-1 and RR2-0.5). As expected, the greater the relative risk threshold, the better is the performance for annual screening in terms of mortality reduction and QALY in the high-risk group. If the high-risk group is defined by a relative risk above 2.0 and consequently screened annually, the mortality reduction is 91.2% in RR2-1 (or 91.9% in RR2-0.5). If the high-risk group is assigned to women with relative risk above 1.5, the mortality reduction is 41.3% in RR1.5–1.0 or (41.4% in RR1.5–0.5). Similarly also for the low-risk group, the mortality reduction depends on the chosen threshold. If the low-risk group is defined by a relative risk below 0.5 and screened triennially, then the mortality reduction is 5.1% with RR 2–0.5 and RR1.5–0.5 (or 5.6% in RR1-0.5). This reflects the lower incidence in this low-risk group, which is 6.9% lifetime incidence.

**Table 3:** Subgroup analysis, performance in clusters.

Strategy	Screening cluster	Population in cluster (%)	Incidence in cluster (%)	Mortality reduction with full adherence	Mortality reduction with 54% adherence	Loss due to low adherence (%)	QALY in full adherence	QALY in 54% adherence	Loss due to low adherence (%)
Routine 3-year	Annual	0.0							
	Biennial	0.0							
	Triennial	100	12.3	–11.2 (–11.4,–11)	–7.1 (–7.2,–6.9)	–37	0.027 (0.027,0.028)	0.017 (0.017,0.017)	–37
Routine 2-year	Annual	0.0							
	Biennial	100	12.3	–14.3 (–14.5,–14)	–8.7 (–8.9,–8.5)	–39	0.037 (0.036,0.037)	0.022 (0.022,0.023)	–41
	Triennial	0.0							
Routine 1-year	Annual	100	12.3	–17.4 (–17.6,–17.1)	–10.3 (–10.5,–10.1)	–41	0.045 (0.045,0.046)	0.027 (0.026,0.027)	–40
	Biennial	0.0							
	Triennial	0.0							
RR 2–1	Annual	1.6	42.0	–91.2 (–95.6,–86.8)	–50.9 (–54.2,–47.6)	–44	0.336 (0.322,0.35)	0.191 (0.18,0.201)	–43
	Biennial	50.6	15.3	–18.7 (–19.1,–18.4)	–11.3 (–11.6,–11)	–40	0.05 (0.049,0.051)	0.03 (0.03,0.031)	–40
	Triennial	47.7	8.6	–7.7 (–7.9,–7.4)	–4.8 (–5,–4.7)	–38	0.017 (0.016,0.017)	0.01 (0.01,0.011)	–41
RR 1–0.5	Annual	46.1	16.7	–25.7 (–26.1,–25.2)	–14.9 (–15.3,–14.6)	–42	0.071 (0.07,0.072)	0.041 (0.041,0.042)	–42
	Biennial	48.5	9.1	–9.8 (–10.1,–9.5)	–6 (–6.2,–5.8)	–39	0.022 (0.022,0.023)	0.013 (0.013,0.014)	–41
	Triennial	5.4	6.7	–5.6 (–6.2,–5)	–3.5 (–4,–3)	–37	0.012 (0.011,0.014)	0.008 (0.007,0.009)	–33
RR 2–0.5	Annual	1.6	42.0	–91.9 (–96.3,–87.5)	–51.3 (–54.6,–48)	–44	0.34 (0.326,0.354)	0.193 (0.182,0.204)	–43
	Biennial	90.0	12.5	–14.6 (–14.8,–14.3)	–8.8 (–9,–8.7)	–40	0.037 (0.037,0.038)	0.023 (0.022,0.023)	–38
	Triennial	8.4	6.9	–5.1 (–5.5,–4.6)	–3.1 (–3.5,–2.7)	–39	0.011 (0.01,0.012)	0.007 (0.006,0.008)	–36
RR 1.5–1.0	Annual	11.9	24.1	–41.3 (–42.4,–40.2)	–23.8 (–24.7,–23)	–42	0.126 (0.123,0.129)	0.073 (0.07,0.075)	–42
	Biennial	42.2	13.7	–16.4 (–16.8,–16.1)	–9.8 (–10.1,–9.5)	–40	0.043 (0.042,0.044)	0.026 (0.025,0.026)	–40
	Triennial	45.9	8.5	–7.6 (–7.9,–7.4)	–4.8 (–5,–4.6)	–37	0.017 (0.016,0.017)	0.01 (0.01,0.011)	–41
RR 1.5–0.5	Annual	11.9	24.1	–41.4 (–42.5,–40.3)	–23.9 (–24.7,–23)	–42	0.127 (0.124,0.13)	0.073 (0.071,0.075)	–43
	Biennial	79.7	11.4	–13.1 (–13.3,–12.8)	–7.9 (–8.1,–7.7)	–40	0.032 (0.032,0.033)	0.019 (0.019,0.02)	–41
	Triennial	8.4	6.9	–5.1 (–5.5,–4.6)	–3.1 (–3.5,–2.7)	–39	0.011 (0.01,0.012)	0.007 (0.006,0.008)	–36

Raising adherence rates in specific risk groups offers promising performance gains. Dividing the total performance gain through the adherence increase gives the average gain for each percent increase. Given biennial routine screening, each increase of 1% in the participation rate results in an increase of 0.85% in mortality reduction and QALY increase of 0.90% on average. However, when increasing the adherence by 1% for women at high risk, e.g., a relative risk higher than 2.0 (1.6% of the population), then this results in an increase in mortality reduction of 0.96% and QALY of 0.93% for this risk group. If the high-risk group is defined by a relative risk of 1.5% and higher, then an increase in adherence by 1% would increase mortality reduction by 0.91% and QALY by 0.91%.

**Fig 2:**
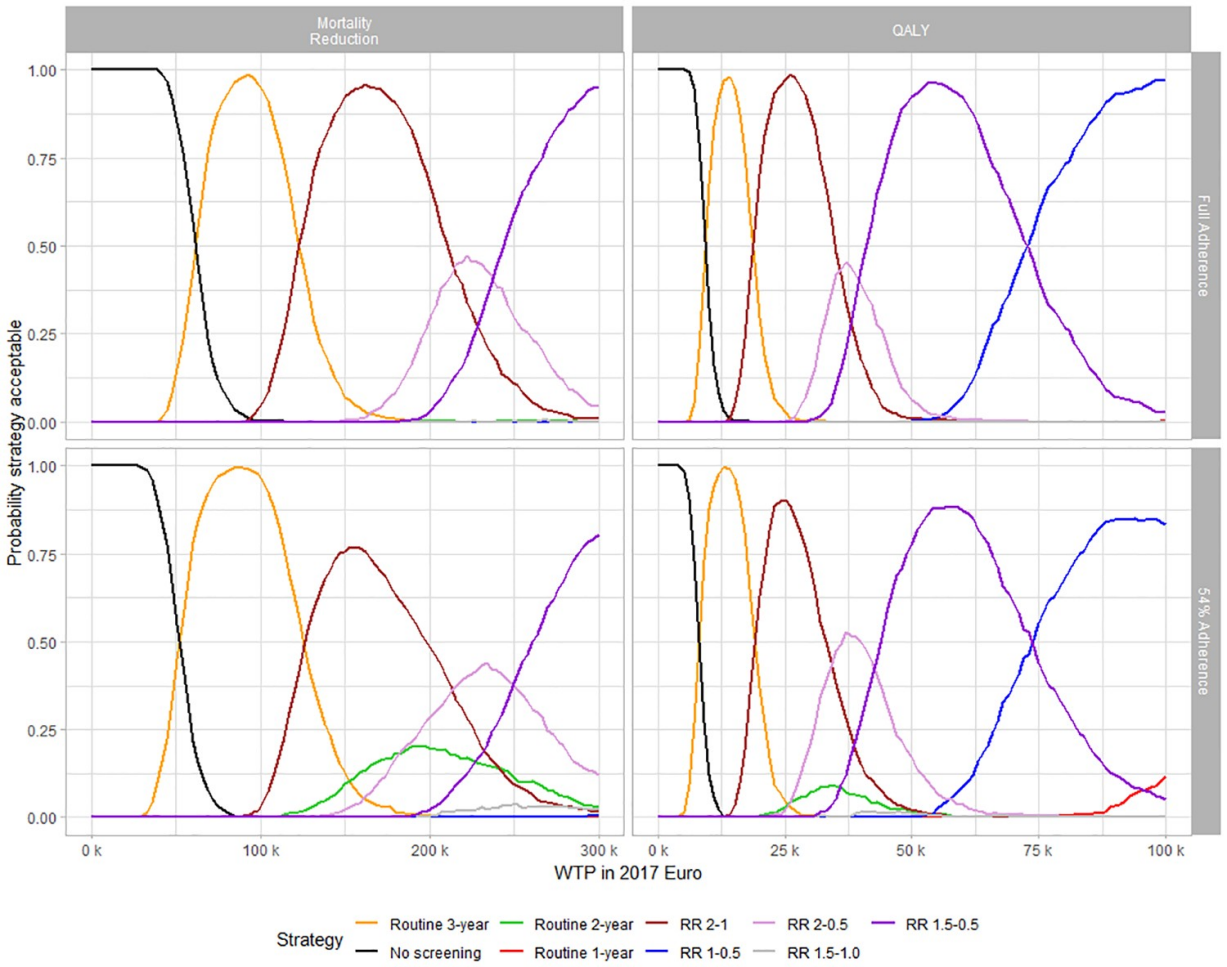
Cost-effectiveness acceptability curves.

From the CEAC Fig 2, we learn that strategy selection depends on the health outcome, the corresponding WTP, and the adherence assumption. If mortality reduction is most important for decision makers, the recommendation should be as follows: at least €54,000 is required to justify the most basic screening strategy, which is routine triennial. This is the case for both full adherence and 54% adherence assumptions, with only small differences. If WTP is over €54,000, the adherence assumption matters. For full adherence, triennial routine screening is the best strategy until WTP reaches €129,000; then RR 2–1 is the best choice for WTP up to €216,000. If adherence is at 54%, however, RR 2–1 intersects with biennial routine screening at €243,000. As RR 2–1 is not always better than routine biennial, it is not clearly economically more efficient. Although there are stratified strategies with higher cost-effectiveness for parts of the WTP scale, there is no strategy clearly dominating biennial routine screening.

If quality of life is the most important criteria, RR 2–1 qualifies as an economically efficient alternative to biennial routine screening. At least €8,000 is required for triennial screening to have a higher probability of being acceptable than no screening. Triennial routine screening is the best alternative for WTP up to €19,000. For higher WTP, the adherence assumption then matters. If full adherence is assumed, RR 2–1 is the best alternative up to WTP of €34,000. For 54% adherence, RR 2–1 remains the most cost-effective strategy until WTP reaches €36,000. In contrast to decisions based on mortality reduction, however, RR 2–1 never intersects with biennial routine screening. For decisions based on quality of life, RR 2–1 is thus more efficient than biennial routine screening.

Looking at the overall uncertainty (the frontier line along the highest CEACs), Fig 2 shows that the probability of having made the right decision is lower with 54% adherence than with full adherence. From the univariate sensitivity analysis in Fig I of the S1 Appendix, we know that the uncertainty is driven by the cost of mammography, the discount rate, the adherence rate, and the incidence of invasive cancers. However, the two most important factors are potentially overdiagnosed cases and QALY losses from screening.

The difference in EVPI between the full adherence and 54% adherence scenarios is €68 per women (assuming an arbitrary WTP of €30,000 per QALY in absence of national estimates). The uncertainty about the adherence assumption thus has substantial monetary implications.

## Discussion

Low participation rates in screening programs are common in many European countries. However, in economic evaluations, low adherence has not received much attention so far. For the economic evaluation of screening strategies, non-adherence affects both cost and effects simultaneously, and thus does not necessarily affect decisions based on the ratio of the incremental cost and effect differences. Thus, a plausible simplification so far has been to disregard low adherence in the economic evaluation. However, we were able to demonstrate in an earlier study that this argument is no longer valid [11]. The results showed that economic recommendations depend on the behavioral assumption underlying non-adherence and the adherence level. Therefore, the aim in the present study was to evaluate economically five different risk-adapted screening strategies and routine screening under the assumption of full adherence vs. the actual adherence rate of 54%.

Our results show that the actual participation rate of 54% substantially reduces the performance of the biennial routine screening program, but also saves 52% of the program cost. At 54% adherence, screening in Germany currently results in a mortality reduction of 9% instead of 16%, which would be possible if full adherence was achieved.

Our analysis shows that risk-stratified screening programs can be more efficient, but the decision is dependent on the decision criteria, i.e., whether mortality reduction or QALY is more important, how much one is willing to pay for these health outcomes, and the adherence assumption. If quality of life is the decision criteria, RR 2–1 is a more efficient alternative to routine biennial screening. Depending on the WTP, though, other stratified screening programs may be preferable alternatives. However, if mortality reduction is more important than quality of life and adherence is 54%, RR 2–1 is not consistently more efficient. In this case, there is no clearly better alternative for all WTP thresholds. The efficiency of risk-stratified screening thus depends on the importance of the decision criteria, the WTP for the criteria, and the actual adherence assumptions.

In addition, the return on investments in screening strategies is affected by adherence. With 54% adherence, the return on investing money in more intensified screening is smaller than expected at full adherence. Every percentage adherence gain is estimated to increase mortality reduction by 0.80% and QALY by 0.85%. Although increasing adherence in the general population could be very difficult and costly, a narrow focus on a selected high-risk population could however produce bigger performance gains. Increasing adherence in women with high relative risk produces even greater increases in mortality reduction and QALY. The value of information analysis demonstrated that adherence gains have substantial monetary implications.

Our analysis has some limitations. First, our model is based on screening performance as observed with analog screen-film mammography. We used this screening technology because the literature used is based on screen-film mammography. This technology is still widely used, but is being replaced by full-field digital mammography. Digital mammography has the advantage of being more accurate in terms of specificity and sensitivity, and thus may improve screening performance [47], especially in women with dense breast tissue [48]. However, the performance increase in women over 50 years is small [49], and there is no evidence that screening adherence differs between analog or digital screening. Second, in the mathematical implementation of the adherence behavior, we relied on published data from the German mammography screening program. In their evaluation reports, they provide detailed information about the recent numbers of invited women and participation rates. However, they do not provide an analysis of the risk within participating and non-participating women. In the absence of this information, we used meta-analyses and systematic reviews describing the existence of a positive association between risk and adherence behavior [50–53] and another systematic review describing the extent of this relationship [53]. However, these studies are not based on the German population. There is evidence suggesting that adherence is also influenced by risk in Germany [54]. Third, this model was originally designed and evaluated in the context of the U.S.A. Our adaptation to the German context utilizes all information and sources that we were aware of. However, there are some elements that could not be adapted. These elements are explicitly the relative risks of developing breast cancer, based on the three risk factors, breast density, family history in a first-degree relative, and previous biopsies, and the cancer stage-specific performance of mammography screening. These three risk factors describe a spectrum of relative risk between 0.33 and 2.88 and thus represent the full risk spectrum associated with women in the general population who are eligible for the mammography screening program in Germany.

Fourth, our model does not incorporate a natural history component of breast cancer. Instead, we rely on the stage-specific detection rates, which implicitly include the natural progression, but do not allow distinction between detected and undetected cancers. Therefore, our model underestimates the effect of mammographic screening, as we only incorporate its effect via the stage shift at diagnosis, but not at earlier diagnosis.

## Conclusion

In this economic evaluation, we compared the cost-effectiveness of risk-stratified screening strategies under the assumption of full adherence and the situation in Germany with a 54% adherence rate. By comparing these two scenarios from the perspective of a decision maker, we aimed to discover whether risk-stratified screening is an economically efficient alternative to routine screening in Germany.

We have shown that a decision for or against risk-stratified screening requires explicit decision criteria. The strategies that maximize mortality reduction do not necessarily also maximize quality of life. In addition, decisions based on perfect assumptions are not the same as decisions based on real-world data. Risk-stratified screening is economically efficient only if adherence is perfect. If adherence is as low as in Germany, stratified screening is not clearly better than routine screening, but dependent on the decision criteria and the actual WTP.

A more promising design for new screening strategies thus consists of combinations of risk stratification mechanisms with interventions to address and improve the low adherence in selected high-risk groups.

## Supporting information

S1 Appendix (DOCX)
