# Supplementary material for: Is risk-stratified breast cancer screening economically efficient in Germany?
Source: PLoS One. 2019 May 23;14(5):e0217213. doi: 10.1371/journal.pone.0217213 (PMC6532918; doi:10.1371/journal.pone.0217213)
Supplement: S1 Appendix — (DOCX) [file pone.0217213.s001.docx]

Risk-stratified breast cancer screening and non-adherence in Germany

Supplementary material

Matthias Arnold ^1,2,3*^, Katharina Pfeifer^4^, Anne S. Quante^4^

^1^ Centre for Health Economics, University of York, York, United Kingdom

^2^ Munich Center of Health Sciences, Ludwig-Maximilians-Universität, Munich, Germany

^3^ Institute of Health Economics and Health Care Management, Helmholtz Zentrum München – German Research Center for Environmental Health, Neuherberg, Germany

**^4^** Frauenklinik, Klinikum Rechts der Isar, Technical University Munich (TUM), Munich, Germany

* Correspondence to:

Matthias Arnold, Centre for Health Economics, University of York, Alcuin ‚A’ Building, York YO105DD, United Kingdom, Tel.: +44-(0)1904 321089, eMail: Matthias.arnold@york.ac.uk

# Model description

The core model is based on a modeling idea and implementation from Schousboe, Kerlikowske (1). We changed the core module to incorporate adaptable screening intervals and non-adherence. A detailed description can be found in an earlier publication [2]. Individual women start in the state “healthy” at the age of 50 years. Over the course of their lifetimes, they may develop invasive cancers, non-invasive ductal carcinoma in situ (DCIS), or die from other causes. Women diagnosed with DCIS have increased likelihood of developing invasive carcinoma later, but may also die first from other causes. If a woman develops invasive cancer, she is diagnosed with one of three cancer stages: local, regional, or distant. Depending on the specific stage of diagnosis, the women dies of breast cancer after some years or remains in the cancer stage until she dies from other causes. This core module can be seen in Fig A.

Fig A. State transition diagram, core module

On top of the survival and mortality module, screening strategies are incorporated by allowing a stage shift from later to earlier disease states. The distributions, which describe whether an invasive cancer is localized, regional, or distant, shift when screening strategies are included. Screening thus does not prevent cancer, but allows earlier diagnosis and longer survival in an earlier cancer stage. This model uses four distributions that reflect cancer stages at the time of diagnosis for women who were not screened, for women with triennial, biennial, or annual screening visits.

Although the screening module follows the logic and design of the original model [1], we added screening adherence and non-adherence as an additional module, which can be used to replicate 100% adherence for validation of other simulation models or allows screening non-adherence at an individual level [2]. The module allows this by random decision making of individuals on their random path through the screening module. At each screening event, a random number is drawn and compared with the individual’s average probability of adhering or not adhering. By drawing random numbers at each event, a random adherence path along the screening path is produced, which reproduces the individual’s average adherence to screening.

# Invasive cancer incidence

We estimated invasive cancer incidence from the publicly available data provided by the German Centre for Cancer Registry Data (ZfKD) [3]. We followed the recommendations of Rosenberg, Check (4) and utilized their web tool to calculate age–period–cohort (APC) models to account for cohort and period trends in cancer incidence data. This method isolates the age-specific incidence rates from shifts in cancer diagnosis by controlling for cohort and period effects: period effects describe time trends that affect all age groups at the same time, whereas cohort effects describe changes in diagnosis or treatment, which have a stronger impact on specific birth cohorts. This method was first propounded by Holford, Cronin (5), later updated by Gangnon, Sprague (6) for application in the U.S.A., and recently applied to Germany by Berkemeyer, Lemke (7). We updated the application to Germany, by utilizing cancer incidence counts for the years 1995 to 2013 provided by the ZfKD [3] and population estimates for the same years provided by the statistical database of the Federal Statistical Office [8]. We used 5-year age groups for breast cancer incidence counts, resulting in 14 age groups from 20–24 years to 85 years plus and structured population estimates for the female population in the same categories. The web-based application of Rosenberg, Check (4) was used to test cohort and period effects on incidence rates. Both cohort and period were found to be statistically significant. Rosenberg, Check (4) recommended using longitudinal age curves, but modulating the incidence rates by the cohort rate ratio. The adjusted age-specific incidence rate reflects both the longitudinal increase in incidence rates and the increased rates of birth cohorts after 1950. Fig B shows the resulting age-specific incidence curve. The curve shows the typical increase between 60 and 75 years, which has been observed in Germany [9] and many other European countries [10].

Fig B. Age-specific incidence rates from APC models, cohort-modulated longitudinal age curve


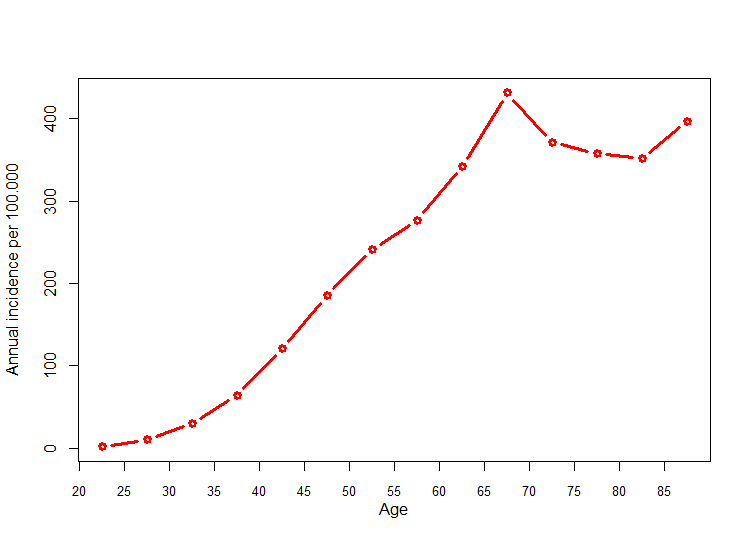


# DCIS incidence

For the incidence of DCIS, we used the same APC methodology as described for invasive cancers. DCIS incidence counts can be publicly accessed from the hospital statistics of the Federal Statistical Office [8] for the years 2000 to 2014. In combination with the population estimates, we could use the same 5-year age groups from 20–24 years to 85 years plus and calculate the APC estimation for DCIS incidence. Similar to the invasive cancer incidence analysis, DCIS also showed significant period and cohort effects, which we accounted for by modulating the longitudinal curve by the cohort ratios. The DCIS incidence curve is shown in Fig C.

Fig C. Age-specific DCIS incidence rates from APC models, cohort-modulated longitudinal age curve


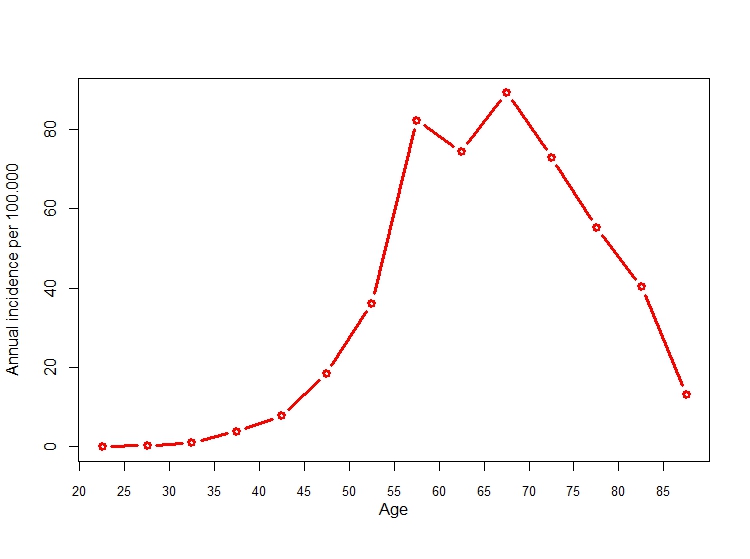


# Risk factors

The risk of developing breast cancer does not only depend on age, but also on a combination of three risk factors (density of breast tissue, history of previous biopsy, family history with breast cancer in a first-degree relative). These risk factors are often used for the general population [1, 11, 12]. The list is obviously not exhaustive, as other important factors include age at menarche, age of first child, usage of hormone replacement therapy, obesity, age at menopause, and others. However, by focusing on these three risk factors, relative risks between 0.41 and 4.65 [13] can be covered, which allows us to simulate women with lower than average risk and women with high risk. Women at very high risk, such as BRCA gene carriers, often have access to intensified surveillance or prophylactic treatments, which are not adequately reflected in this model.

Relative risks were calculated as the risk within the risk exposed group over the risk in the unexposed group ${RR}_{RiskGroup}=\frac{\frac{{Cases}_{Risk carriers}}{{Population}_{Risk carriers}}}{\frac{{Cases}_{Risk non-carriers}}{{Population}_{Risk non-carriers}}}$. For the three risk factors here, prevalence and relative risk scores were approximated from the literature for the German population. The relative risk of breast density lies between 0.388 and 1.675 depending on the breast imaging reporting and data system (BIRADS) categorization of breast density levels and the age of the woman [1, 14] (Table A). Research suggests that breast density decreases with age [15, 16]. In order to reflect the natural decrease in breast density, we allowed breast density to change every 5 years. Based on the breast density prevalence reported for Germany by Weigel, Heindel (17) and the probability of changing breast density categories reported for the U.S.A, by Kerlikowske, Ichikawa (16), we calculated the age-specific distribution of breast density using the BIRADS categories (Table B).

Table A. Relative risk due to breast density

| **Age group (years)** | **BIRADS 1** | **BIRADS 2** | **BIRADS 3** | **BIRADS 4** |
| --- | --- | --- | --- | --- |
| **50–59** | 0.388 | 0.807 | 1.251 | 1.623 |
| **60–64** | 0.400 | 0.832 | 1.291 | 1.675 |
| **65–69** | 0.581 | 0.885 | 1.228 | 1.283 |
| **70+** | 0.600 | 0.914 | 1.268 | 1.325 |
|  | | | | |

Table B. Age-specific BIRADS distribution (%)

| **Age group (years)** | **BIRADS 1** | **BIRADS 2** | **BIRADS 3** | **BIRADS 4** |
| --- | --- | --- | --- | --- |
| **50–54** | 6.1 | 38.7 | 48.3 | 6.9 |
| **55–59** | 7.4 | 45.5 | 41.3 | 5.8 |
| **60–64** | 8.6 | 48.5 | 37.9 | 5.0 |
| **65–69** | 9.5 | 50.0 | 36.1 | 4.5 |
| **70+** | 10.0 | 50.9 | 35.0 | 4.2 |

For family history in a first-degree relative and previous biopsies, we used estimates from Schousboe, Kerlikowske (1) and Tice, Cummings (18) based on the Breast Cancer Surveillance Consortium [13]. The relative risk of invasive cancer is 1.454 or 0.938 in the presence or absence of a family history and 1.495 or 0.906 in the presence or absence of a previous biopsy. Some 16% of all woman have a family history of breast cancer, and 28% underwent a biopsy to clarify abnormal tissue [14].

# Mammography screening and adherence

We assess mammography screening for women with average and high risk aged between 50 and 69 years. Women at very high risk, such as BRCA1/2 gene carriers, have access to intensified screening programs, which are not considered in this study. Owing to ongoing discussion about the balance between mammography benefits and its harmful effects, a stratified approach to mammography is considered. The underlying idea is that routine biennial screening in women with low risk is less effective than screening higher risk women. However, harmful screening effects, false-positive results and potential overtreatment, however are the same for both groups. Separating low from high-risk and screening high-risk more intensively, but low-risk women less often might thus improve the balance.

A common risk factor that can be used for stratification, is breast density. High density is not only a risk factor for invasive breast cancer, but is also a natural obstacle for mammographic imaging [19]. Mammography screening is less sensitive in dense breast tissue [17] and thus shorter intervals might help to detect breast cancers earlier [11].

Schousboe, Kerlikowske (1) and Kerlikowske, Zhu (20) described the outcomes of mammography screening stratified for breast density and age from data from the American mammography program [14]. As described in the supplementary material of an earlier publication [2], we utilized their analyses to accommodate triennial, biennial and annual screening intervals. With increasing screening frequency, the likelihood of being diagnosed with earlier rather than late cancers is also increased. In addition, with increasing age and increasing breast density, late cancers become more frequent. Since the introduction of the national breast cancer screening program in 2009, 90% of women have been invited to biennial mammography screening [21]. However, as of 2014 only 54% of eligible women participated. The German screening program thus does not fulfil the European goal of 70% participation [22]. Accordingly, we compare routine screening and risk-stratified screening in two scenarios of 54% adherence and full adherence. In the case of non-adherence, we assume that the woman loses the protective effect of screening, has a higher likelihood of being diagnosed with late cancers, and has the same stage distribution as women who do not participate in screening. The less favorable distribution is maintained until she participates again.

We assume, that at the moment of the first screening, each woman is confronted with her risk level and is assigned a random probability of adhering to screening based on risk score. For the age group of the 50-year-old women, there are 16 combinations of risk factors that can be used to calculate 10 year risk scores [14].

Table C presents the three adherence scenarios, the corresponding risk levels and the expected participation rate assuming the prevalence of risk factors as in Schousboe, Kerlikowske (1). In order to test these estimates in the probabilistic sensitivity analysis, the values were randomly changed to reflect a 10% spread around the mean value.

Table C. Adherence level scenarios and risk score

| Breast density (in BIRAD levels) | Family history (y/n) | Previous biopsy (y/n) | | 10-year risk of developing invasive breast cancer [18] (mean is 1.46) | Adherence probability (%) |
| --- | --- | --- | --- | --- | --- |
| 1 | 0 | 0 | | 0.97 | 44.50 |
| 1 | 0 | 1 | | 1.55 | 48.94 |
| 1 | 1 | 0 | | 1.40 | 47.97 |
| 2 | 0 | 0 | | 2.22 | 52.33 |
| 1 | 1 | 1 | | 1.80 | 50.35 |
| 2 | 0 | 1 | | 2.85 | 54.70 |
| 3 | 0 | 0 | | 2.57 | 53.72 |
| 2 | 1 | 0 | | 4.07 | 58.07 |
| 4 | 0 | 0 | | 2.69 | 54.15 |
| 3 | 0 | 1 | | 4.26 | 58.50 |
| 2 | 1 | 1 | | 3.84 | 57.52 |
| 4 | 0 | 1 | | 6.05 | 61.82 |
| 3 | 1 | 0 | | 3.23 | 55.88 |
| 4 | 1 | 0 | | 5.10 | 60.20 |
| 3 | 1 | 1 | | 4.60 | 59.23 |
| 4 | 1 | 1 | | 7.23 | 63.50 |
| Expected participation rate | | | 54.3% | | |

# Screening strategies

We incorporated three routine screening strategies, with triennial, biennial, or annual intervals, and compared them with five risk-stratified strategies. The first stratified strategy was proposed by Trentham-Dietz, Kerlikowske (11) (TDK) for the American health system. In an earlier study, we compared their proposal against two risk-stratified strategies [1, 12] and found that the TDK strategy is superior or highly cost-effective compared with biennial routine screening or the other two stratified proposals. In this study, we analyze the TDK strategy in the German setting, but also propose four alternative strategies, which are specifically designed for the risk profile of the German population described earlier. We propose four strategies stratified by relative risk. In Table D the five stratified strategies are shown with their suggested screening intervals for each combination of risk factors and age.

Table D. Stratified screening, intervals by risk factors and age

| **Strategy** | **Age (years)** | **BIRADS 1** | | | | | **BIRADS 2** | | | | | **BIRADS 3** | | | | | **BIRADS 4** | | | |  |
| --- | --- | --- | --- | --- | --- | --- | --- | --- | --- | --- | --- | --- | --- | --- | --- | --- | --- | --- | --- | --- | --- |
|  |  | 0 | FH | Bio | both | 0 | | FH | Bio | both | 0 | | FH | Bio | both | 0 | | FH | Bio | both | |
| **TDK [11]** | 50–59 | 3 | 3 | 3 | 2 | 3 | | 3 | 3 | 2 | 2 | | 2 | 2 | 1 | 2 | | 2 | 1 | 1 | |
|  | 60–64 | 3 | 3 | 3 | 2 | 3 | | 3 | 3 | 2 | 2 | | 2 | 2 | 1 | 2 | | 2 | 1 | 1 | |
|  | 65–70 | 3 | 3 | 3 | 2 | 3 | | 3 | 3 | 2 | 2 | | 2 | 2 | 1 | 2 | | 2 | 1 | 1 | |
|  | | | | | | | | | | | | | | | | | | | | | |
| **RR 2-1** | 50–59 | 3 | 3 | 3 | 3 | 3 | | 2 | 2 | 2 | 2 | | 2 | 2 | 1 | 2 | | 1 | 1 | 1 | |
|  | 60–64 | 3 | 3 | 3 | 3 | 3 | | 2 | 2 | 2 | 2 | | 2 | 2 | 1 | 2 | | 1 | 1 | 1 | |
|  | 65–70 | 3 | 3 | 3 | 2 | 3 | | 2 | 2 | 2 | 2 | | 2 | 2 | 1 | 2 | | 2 | 2 | 1 | |
|  | | | | | | | | | | | | | | | | | | | | | |
| **RR 1-0.5** | 50–59 | 3 | 2 | 2 | 2 | 2 | | 1 | 1 | 1 | 1 | | 1 | 1 | 1 | 1 | | 1 | 1 | 1 | |
|  | 60–64 | 3 | 2 | 2 | 2 | 2 | | 1 | 1 | 1 | 1 | | 1 | 1 | 1 | 1 | | 1 | 1 | 1 | |
|  | 65–70 | 3 | 2 | 2 | 1 | 2 | | 1 | 1 | 1 | 1 | | 1 | 1 | 1 | 1 | | 1 | 1 | 1 | |
|  | | | | | | | | | | | | | | | | | | | | | |
| **RR 2-0.5** | 50–59 | 3 | 2 | 2 | 2 | 2 | | 2 | 2 | 2 | 2 | | 2 | 2 | 1 | 2 | | 1 | 1 | 1 | |
|  | 60–64 | 3 | 2 | 2 | 2 | 2 | | 2 | 2 | 2 | 2 | | 2 | 2 | 1 | 2 | | 1 | 1 | 1 | |
|  | 65–70 | 3 | 2 | 2 | 2 | 2 | | 2 | 2 | 2 | 2 | | 2 | 2 | 1 | 2 | | 2 | 2 | 1 | |
|  | | | | | | | | | | | | | | | | | | | | | |
| **RR 2** | 50–59 | 2 | 2 | 2 | 2 | 2 | | 2 | 2 | 2 | 2 | | 2 | 2 | 1 | 2 | | 1 | 1 | 1 | |
|  | 60–64 | 2 | 2 | 2 | 2 | 2 | | 2 | 2 | 2 | 2 | | 2 | 2 | 1 | 2 | | 1 | 1 | 1 | |
|  | 65–70 | 2 | 2 | 2 | 2 | 2 | | 2 | 2 | 2 | 2 | | 2 | 2 | 1 | 2 | | 2 | 2 | 1 | |
| **0: No additional risk factors**  **FH: family history in first-degree relative**  **Bio: history of previous biopsy**  **Both: both risk factors** | | | | | | | | | | | | | | | | | | | | | |

# Diagnostic work-up

The German mammography screening program provides annual evaluation reports from which diagnostic work-up rates can be extracted. In 2014, 4.3% of all women were invited for diagnostic work. Work-up rates were higher for women at first screening (10%) than for women at repeated screenings (3%) [22]. Some 1.1% of all screened women had an indication for biopsy [23]. Accordingly, 25% of all women invited for diagnostic work-up had an indication for biopsy. From 32,988 women with indication for biopsy, 16,632 (50.4%) were diagnosed with breast cancer. Accordingly, among those women undergoing biopsy, 49.6% were not diagnosed with breast cancers. In the annual evaluation reports, these biopsies are considered unnecessary [23]. The ratio of benign over malign biopsy results (or unnecessary over necessary biopsies) differs for the type of biopsy and for first screening or later screenings. In Germany, either core biopsy with ultrasound or X-ray guidance or vacuum biopsy with X-ray guidance is used. From the annual quality report 2014 [23], we know that 19,719 (60%) core biopsies with ultrasound guidance, 13,038 (39%) vacuum biopsies with X-ray guidance, and 284 (1%) X-ray-guided core biopsies were used. For core biopsy (either ultrasound or X-ray guided), the ratio is 48.2% for first screening and 80.1% for later screenings. For vacuum biopsy, the ratio is 24.3% for first screening and 44.9% for later screenings.

From this, we can extract the false-positive rates for recall screening and biopsies and for first screening examinations and later screenings. For first screening examinations, 7.5% have a false-positive screening result and receive unnecessary recall screening; 0.97% receive a false-positive recall, of whom 0.73% receive unnecessary core biopsy and 0.24% receive unnecessary vacuum biopsy. For later screening examinations, 2.25% have a false-positive screening result and undergo unnecessary recall screening; 0.5% receive a false-positive recall, of whom 0.37% receive unnecessary core biopsy and 0.13% receive unnecessary vacuum biopsy.

# Breast cancer mortality

Cancer mortality is based on relative survival estimates from the Munich Cancer Registry [24]. They provide tumor-specific survival analyses based on TNM stages for 3.84 million people in Bavaria. We converted TNM stages into summary stages with the following conversion system (Table E). The conversion is based on the definition of the summary stages [25]. Local cancers are restricted to the original area, regional cancers have affected lymph nodes, other tissue, or organs, and distant carcinomas have metastasized. This conversion system was also used by other researchers [26]. Local cancers are T1–3 and N0, regional cancers are either T4 or T1–3 and N1, and distant cancers are M1. Tumorregister München (24) provides survival data for each TNM stage over 20 years. Using the summary stages, we produced aggregate survival rates from the combination of TNM-specific survival case numbers. We assumed that patients surviving more than 20 years would not die from cancer. Fig D shows the corresponding survival curves.

Table E. Stage conversion

|  | **T1N0M0** | **T2N0M0** | **T3N0M0** | **T4N0M0** | **T1N+M0** | **T2N+M0** | **T3N+M0** | **T4N+M0** | **M1*** |
| --- | --- | --- | --- | --- | --- | --- | --- | --- | --- |
| **local** | 1 | 1 | 1 |  |  |  |  |  |  |
| **regional** |  |  |  | 1 | 1 | 1 | 1 | 1 |  |
| **distant** |  |  |  |  |  |  |  |  | 1 |
| *** For metastatic cancers independent of primary tumor size and lymph nodes** | | | | | | | | | |

Tumorregister München (24) provides survival data for each TNM stage over 20 years. Using the summary stages, we produced aggregate survival rates from the combination of TNM-specific survival case numbers.

Fig D. Survival curves, based on relative survival reported by the Munich Cancer Registry


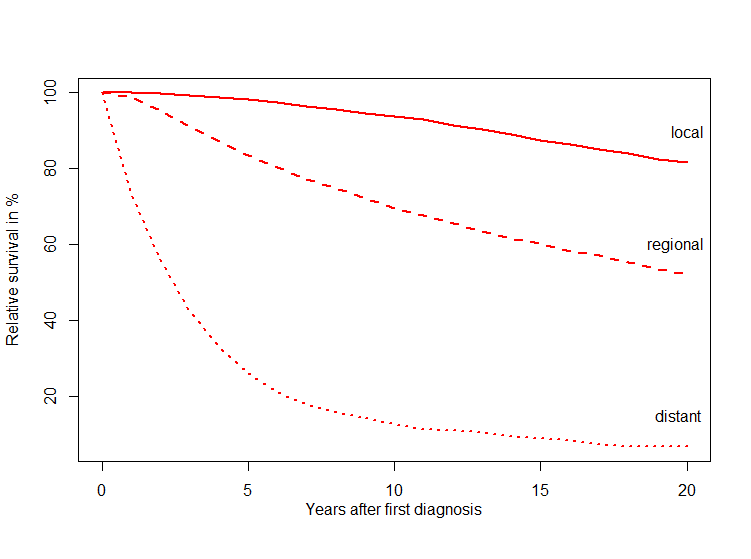


# Background mortality

Following the guidance of Rosenberg (27) for calculating background mortality, we adjusted mortality rates for breast cancer mortality in order to extract mortality from other causes. This method is used consistently in and recommended by the CISNET working group [28]. We used the life tables of 2015 to calculate age-specific all-cause mortality rates for the German population [8]. As the life tables also include mortality resulting from breast cancer, we adjusted the mortality statistics for breast cancer using the cause of death statistics [8]. As the all-cause mortality rates are age specific for every life year, but death statistics are age specific for 5-year intervals, we fitted a polynomial function as seen in Fig E.

Fig E. Age-specific breast mortality as percentage of all-cause mortality


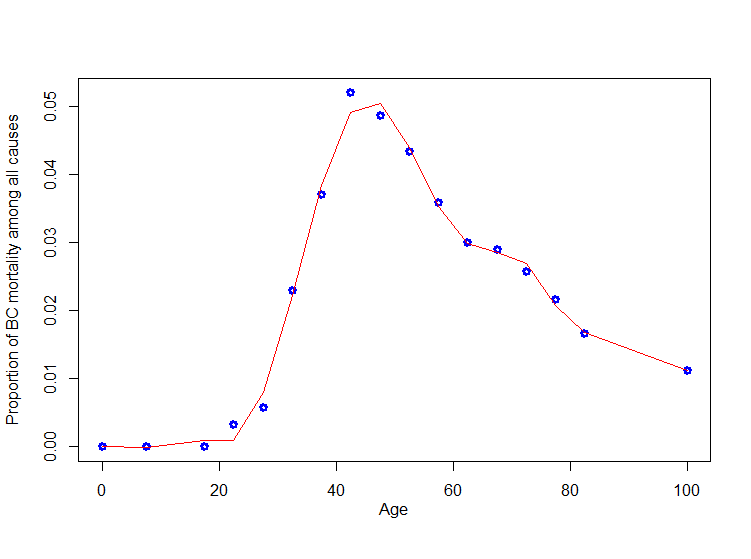


Distribution fitting was based on the Akaike and Bayesian information criteria as well as graphical validation using QQ plots. After reducing age-specific all-cause mortality by the fraction of breast cancer deaths, the remaining mortality probabilities can be interpreted as background mortality in the absence of breast cancer. Fig F illustrates the difference between background and all-cause mortality. Despite the fact that the highest fraction of breast cancer deaths appears between 40 and 60 years, a “dent” in the mortality probability is only noticeable in the last years of life when overall mortality is much higher.

Fig F. All-cause mortality and other-than-BC mortality


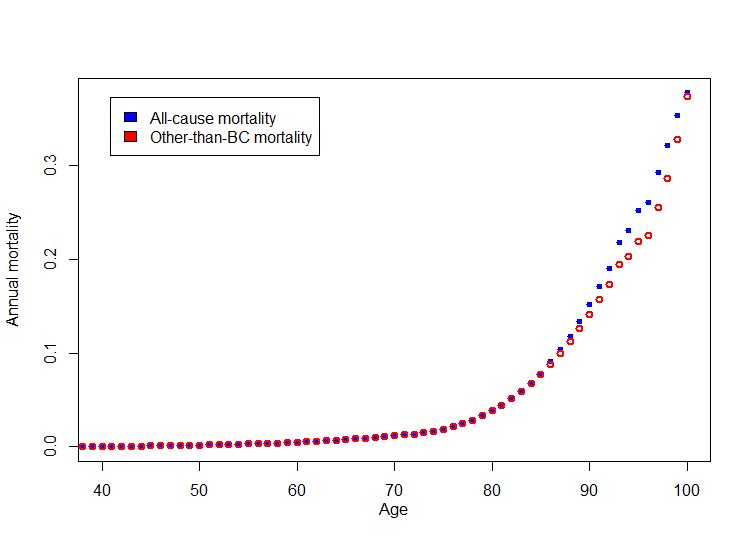


# Cost parameters

For screening and diagnostic work-up, we used the price catalog of the Kassenärztliche Bundesvereinigung (KBV) for outpatient services paid by statutory health insurances in Germany [29] (Table F). In the KBV pricing system, recall screening and core biopsy are combined. Accordingly, the additional cost of biopsy after recall screening consists only of the histopathological evaluation and the supplement for vacuum biopsy.

Table F. EBM prices for screening and diagnostic work-up

| **Phase of care** | **Provider group** | **Code** | **Description** | **Price in 2017 Euro** |
| --- | --- | --- | --- | --- |
| **Mammography screening** | Program responsible | 01751 | Patient briefing | 6.63 |
|  | Program responsible | 01750 | Mammography screening | 58.23 |
|  | Program responsible / 1. Evaluator | 01752 | Consolatory evaluation of mammographic images | 4.32 |
|  | 2. Evaluator | 01752 | Consolatory evaluation of mammographic images | 4.32 |
| **Diagnostic work-up** | Program responsible | 01753 | Clarification diagnosis I: Obligatory additional imaging, facultative core biopsy using either X-ray or sonography guided | 94.45 |
|  | Program responsible / Biopsy physician | 01759 | Supplement for vacuum biopsy | 29.58 |
|  | Pathologist | 01756 | Histopathological evaluation of biopsy material, for each of three specimens | 10.21 |
|  | Pathologist | 01757 | Supplement for histopathological evaluation, for each of three specimens* | 10.89 |
| **Aggregates** | Mammographic screening | | | 73.50 |
|  | Additional cost for recall screening after mammography | | | 94.45 |
|  | Additional cost for core biopsy after recall screening | | | 21.10 |
|  | Additional cost for vacuum biopsy after recall screening | | | 39.79 |
| ***** X-ray guided core-needle biopsies require up to six specimens [30, 31]. | | | | |

Treatment costs were extracted from the literature and confirmed by clinical experts. As treatment regimens depend on the molecular subtype of cancer, we used three subtypes, triple-negative (T–), hormone receptor positive (HR+), and HER2/neu positive (HER2/neu), with corresponding treatment proportions for each. Treatment costs for these molecular subtypes were analyzed by Muller, Danner (32) for local and metastatic carcinoma in BRCA-positive women with data from the German Consortium for Hereditary Breast and Ovarian Cancer [33]. We adjusted the proportion of the molecular subtypes in order to reflect the distribution in women over 50 years from the literature [34]. From a gene database of 4467 breast cancer patients, Liedtke, Rody (34) estimated that the three subtypes (T–, HR+, HER2/neu) can be found in 21%, 7.3%, and 71.7% of all women over 50 years.

We updated prices to 2017 using the consumer price index [8]. For the ratio of breast-conserving therapy and mastectomy, we used data from the Munich Cancer Registry reporting conducted surgeries for each category of primary tumor (T1 to T4) [24]. We assumed Tis to correspond to DCIS, T1 and T2 to local cancers, and T3/4 to regional cancers. We further assumed that distant cancers share this relation with regional cancers, knowing that distant carcinomas are 9.75 times more likely to appear with T3/4 than with T1 or T2 [24]. With the same grading conversion, we assigned the proportions of radiation therapy after breast-conserving therapy or mastectomy.

We utilized the German S3 guideline to extract the probability of chemotherapy in post-menopausal women with local cancers [35]. Chemotherapy is recommended for HER2/neu and T– tumors. Endocrine-sensitive women are supposed to be treated preferably with endocrine therapy only if tumor grade is 1 or 2. Accordingly, we used the probability of tumor grade 3 and 4 in local tumors from the Munich Cancer Registry [24] to determine the probability of chemotherapy in hormone receptor-positive women. As Muller, Danner (32) only described local and distant cancers, we used additional literature to incorporate regional cancers. As regional cancer patients are nodal positive in the majority of cases [24] and the German S3 guideline recommends chemotherapy for all nodal-positive patients except Luminal A [35], we assumed full chemotherapy coverage for T– and HER2/neu and used the distribution of Luminal A in nodal-positive patients [34] to generate the proportion of chemotherapy in HR+ regional cancers. Figs G and H illustrate the assumptions underlying the treatment decisions, Table F shows the treatment cost.

HR+ and HER2/neu patients may receive endocrine therapy. We assumed that women over 50 years are mainly post-menopausal and thus receive upfront aromatase inhibitors as endocrine therapy (Anastrozole, Letrozole, or Exemestan). We used the probabilities of endocrine treatment from the literature [36, 37] and DMP quality reports (reported in Table F), [38] cost estimates from Lux, Wockel (39), in which we adjusted the prices to current standards for Anastrozole, Letrozole, and Exemestan [40], and the distribution of the three agents as reported by Schwabe and Paffrath (41).

Fig G. Treatment decision – surgery and radiotherapy


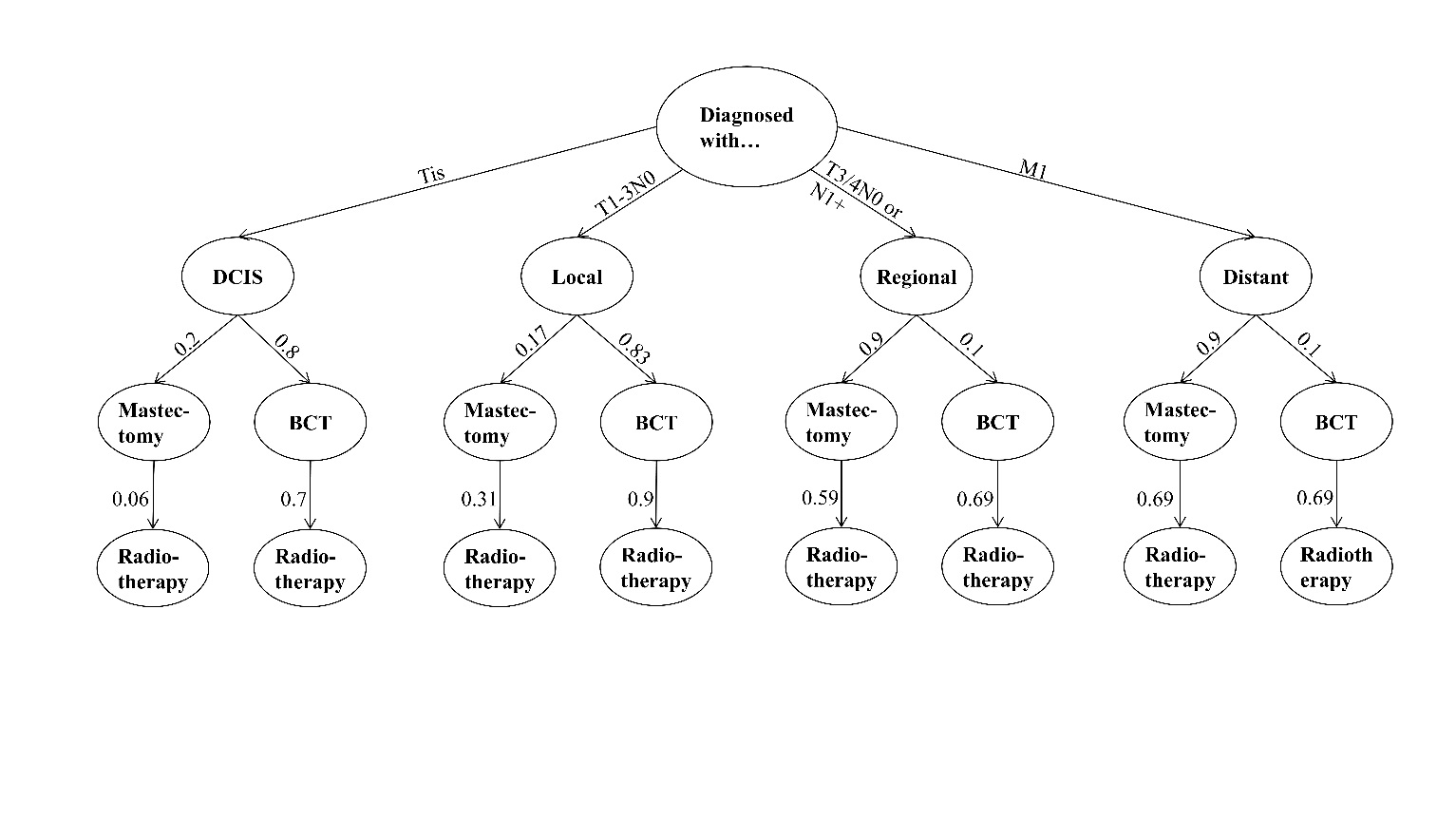


Fig H. Treatment decision – chemotherapy


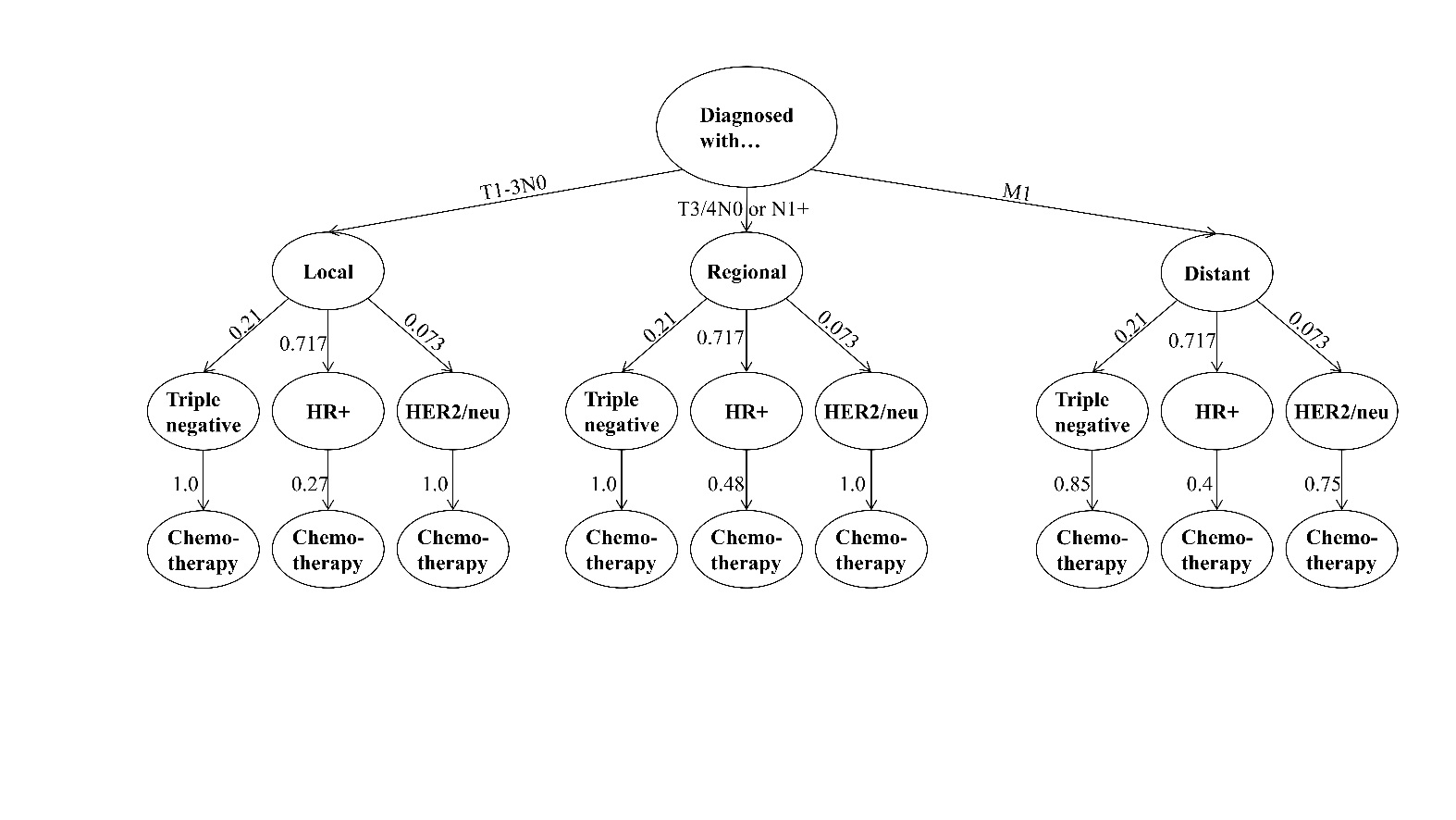


Table G. Treatment cost

| **Treatment phase** | **Cost in 2017 Euro (SD)** | **Proportion (SE)** | **Source** |
| --- | --- | --- | --- |
| Psychological treatment after cancer diagnosis | 1,252 (500) | 0.36 (0.06) | [32] |
| Surgical treatments |  |  |  |
| DCIS |  |  |  |
| Breast-conserving therapy | 4,391 (1,756) | 0.80 | [24, 32] |
| Mastectomy | 6,667 (2,667) | 0.20 | [24, 32] |
| Local cancers |  |  |  |
| Breast-conserving therapy | 4,391 (1,756) | 0.83 | [24, 32] |
| Mastectomy | 6,667 (2,667) | 0.17 | [24, 32] |
| Regional cancers |  |  |  |
| Breast-conserving therapy | 4,391 (1,756) | 0.10 | [24, 32] |
| Mastectomy | 6,667 (2,667) | 0.90 | [24, 32] |
| Distant cancers |  |  |  |
| Breast-conserving therapy | 4,391 (1,756) | 0.10 | [24, 32] |
| Mastectomy | 6,667 (,2667) | 0.90 | [24, 32] |
| Chemotherapy |  |  |  |
| Local cancers |  |  |  |
| T– (21%) | 6,479 (2,591) | 1.00 | [32, 35] |
| HR+ (71.7%) | 6,479 (2,591) | 0.27 | [24, 32, 35] |
| HER2/neu (7.3%) | 26,988 (10,795) | 1.00 | [32, 35] |
| Regional cancers |  |  |  |
| T– (21%) | 6,479 (2,591) | 1.00 | [32, 35] |
| HR+ (71.7%) | 6,479 (2,591) | 0.48 | [32, 34, 35] |
| HER2/neu (7.3%) | 26,988 (10,795) | 1.00 | [32, 35] |
| Distant cancers |  |  |  |
| T– (21%) | 19,488 (7,928) | 0.85 | [32] |
| HR+ (71.7%) | 12,408 (4,963) | 0.40 | [32] |
| HER2/neu (7.3%) | 49,604 (19,841) | 0.75 | [32] |
| Chemotherapy-induced events and treatments |  |  |  |
| Neutropenic sepsis | 5,880 (2,352) | 0.15 (0.04) of patients with chemotherapy | [32] |
| Neulasta (pegfilgrastim) | 10,019 (4,008) | 0.50 (0.13) of patients with chemotherapy | [32] |
| Antiemetics | 503 (201) | All patients with chemotherapy | [32] |
| Bisphosphonates | 428 (171) | All patients with distant cancers | [32] |
| Endocrine therapy for HR+ and HER2/neu |  |  |  |
| Local cancers | First year: 1118 (605)  Years 2–5: 717 (381) | 0.90 (0.11) | [38, 39, 41] |
| Regional cancers | First year: 1118 (605)  Years 2–5: 717 (381) | 0.62 (0.11) | [36, 39, 41] |
| Distant cancers | First year: 1118 (605)  Years 2–5: 717 (381) | 0.49 (0.11) | [37, 39, 41] |
| Radiotherapy |  |  |  |
| After breast-conserving therapy |  |  |  |
| DCIS | 1,821 (728) | 0.70 | [24, 32] |
| Local | 1,821 (728) | 0.90 | [24, 32] |
| Regional | 1,821 (728) | 0.69 | [24, 32] |
| Distant | 1,821 (728) | 0.69 | [24, 32] |
| After mastectomy |  |  |  |
| DCIS | 1,821 (728) | 0.06 | [24, 32] |
| Local | 1,821 (728) | 0.31 | [24, 32] |
| Regional | 1,821 (728) | 0.59 | [24, 32] |
| Distant | 1,821 (728) | 0.69 | [24, 32] |
| Lymph drainage/physiotherapy | 1,505 (602) | 0.25 (0.06) | [32] |
| Palliative care for patients dying of breast cancer | 11,334 (4,534) | All women dying of cancer | [32] |

# Utility parameters

We used EQ-5D utility parameters for health-related quality of life (HRQoL) measurement (Table H). In the absence of German EQ-5D estimates for breast cancer patients, we used utility weights derived from a Swedish breast cancer study conducted by Lidgren, Wilking (42) using the U.K. tariff [43]. Schousboe, Kerlikowske (1) calculated age-specific breast cancer weights from the Swedish study using age-specific EQ-5D estimates for the general population from Burström, Sun (44) and extrapolating the HRQoL losses for age-specific DCIS, local, regional, and distant disease states.

Table H. EQ-5D value set for breast cancer, based on Swedish breast cancer patients

| **Age (years)** | **Healthy** | **DCIS** | | **Local** | | **Regional** | | **Distant** | |
| --- | --- | --- | --- | --- | --- | --- | --- | --- | --- |
|  |  | First year | Later years | First year | Later years | First year | Later years | First year | Later years |
| **40–49** | 0.859 | 0.777 | 0.859 | 0.727 | 0.842 | 0.647 | 0.777 | 0.647 | 0.715 |
| **50–59** | 0.845 | 0.764 | 0.845 | 0.715 | 0.828 | 0.636 | 0.765 | 0.636 | 0.703 |
| **60–69** | 0.812 | 0.734 | 0.812 | 0.687 | 0.796 | 0.611 | 0.735 | 0.611 | 0.676 |
| **70–79** | 0.788 | 0.712 | 0.788 | 0.667 | 0.772 | 0.593 | 0.713 | 0.593 | 0.656 |
| **80+** | 0.762 | 0.689 | 0.762 | 0.645 | 0.747 | 0.574 | 0.69 | 0.574 | 0.634 |

Both mammography screening and diagnostic work-up may affect quality of life. However, research on the effects of mammography screening on quality of life is not yet conclusive. Expert interviews in the Netherlands using a time-trade-off method estimated screening to reduce HRQoL by 0.006 for 1 week [45]. A study using the same method, but with American women enrolled in the mammography program, reported HRQoL losses from screening of 0.196 for 2 weeks [46]. Tosteson, Fryback (47) reported short-term anxiety from mammography screening, which did not translate into HRQoL decrements. This could indicate either that short-term anxiety does not significantly affect HRQoL or that the EQ-5D is not sensitive enough. In order to reflect the uncertainty, we assumed that short-term anxiety would not reduce HRQoL significantly. However, we allowed short-term utility losses of up to 0.196 for 2 weeks in the sensitivity analysis.

For diagnostic work-up involving biopsy, there is evidence of significant effects on HRQoL [48-51]. Verkooijen, Buskens (48) found that core biopsy implies EQ-5D utility scores of 0.73 1 day before biopsy and 0.71 4 days after biopsy. They did not collect long-term effects on HRQoL. However, Maxwell, Bugbee (52) reported that HRQoL as pain and anxiety returned to normal levels within 30 days. This implies HRQoL losses of 0.093 on the first day, 0.113 on day 4, and normalization after 30 days. We used linear interpolation for the days in between. In a sensitivity analysis, we used the expert-based time-trade-off estimate of 0.447 for 2 weeks for both types of biopsy [45].

Vacuum-assisted breast biopsy (VABB) is often experienced as a more painful procedure [53, 54], Domeyer, Sergentanis (49) found that the anxiety accompanying VABB may already reduce HRQoL even before the actual event. Even 18 months after VABB, long-term effects associated with pain were observed. Using the EQ-5D index, they estimated utility scores of 0.729 just before the biopsy, 0.787 4 days after biopsy, and 0.769 up to 18 months later. VABB thus reduces HRQoL in the short term by 0.1 on the first day, 0.04 by day 4, and 0.06 after 18 months. For the days in between, we used linear interpolation. In a sensitivity analysis, we assumed that the higher HRQoL decrement persists until the next observation. Table I provides an overview of the HRQoL parameters and the sensitivity ranges.

Table I. HRQoL parameters for screening and diagnostic work-up

| **Treatment or method** | **Base parameter** | | | **Sensitivity range** | | |
| --- | --- | --- | --- | --- | --- | --- |
|  | Utility weight | Duration or time | HRQoL loss over 1 year | Utility weight | Duration | HRQoL loss over 1 year |
| **Mammography screening** | 0 | n.a. | 0 | 0  0.196 | n.a.  2 weeks | 0  0.0075 |
| **Core needle biopsy** | 0.093 | 1 day before | 0.005 | 0 | n.a. | 0 |
|  | 0.113 | Day 4 |  | 0.447 | 2 weeks | 0.017 |
|  | 0 | Day 30 |  |  |  |  |
| **Vacuum biopsy** | 0.1 | 1 day before | Year 1: 0.047  Year 2: 0.027 | 0  0.1 | n.a.  4 days | 0  Year 1: 0.061 |
|  | 0.04 | Day 4 |  | 0.04 | Day 4 | Year 2: 0.030 |
|  | 0.06 | After 18 months |  | 0.06 | 18 months minus 5 days |  |
|  | 0 | After 18 months +1 day |  |  |  |  |

# Distributions and ranges for sensitivity analysis

As mentioned frequently throughout the technical appendix, we use standard deviations and ranges for most of the point estimates used for parameter assumptions. We test the uncertainty surrounding these point estimates in a probabilistic sensitivity analysis, which randomly selects variation of these point estimates based on distribution functions. We chose commonly used distribution functions for our parameters, such as normal distribution for epidemiologic parameters, gamma distribution for cost parameters and utility parameters, and uniform distribution for parameters using fixed parameter ranges. Table J summarizes these parameters, point estimates, standard deviations, and distributions.

Table J. Parameters, distributions, and ranges

| **Parameter** | **Distribution** | **Standard deviation** | **Range** | **Source** |
| --- | --- | --- | --- | --- |
| Screening and diagnostic work-up cost parameter | Gamma | 10% of mean | | Assumption |
| Treatment cost parameters | Gamma | See Table G | | [32, 39] |
| QALY loss from treatment | Gamma | 10% of mean |  | Assumption |
| QALY loss from screening and diagnostic work-up | Uniform | See Table I | | [45, 46, 49] |
| Invasive cancer incidence | Normal | 10% of mean |  | Assumption |
| Invasive cancer mortality | Normal | 10% of mean |  | Assumption |
| DCIS incidence | Normal | 10% of mean |  | Assumption |
| Overdiagnosis | Uniform |  | 0–10% | [1] |
| Adherence probability | Uniform | 5% of mean |  | Calculation based on [23] |
|  |  |  |  |  |

# Tornado diagram for univariate sensitivity analysis

Fig I. Tornado diagrams

|  |  |
| --- | --- |
|  |  |
|  |  |
|  |  |

# References

1. Schousboe JT, Kerlikowske K, Loh A, Cummings SR. Personalizing Mammography by Breast Density and Other Risk Factors for Breast Cancer: Analysis of Health Benefits and Cost-Effectiveness. Annals of internal medicine. 2011;155(1):10-20.

2. Arnold M, Quante AS, editors. Personalized mammography screening and screening adherence - A simulation of cost-effectiveness. ISPOR 19th Annual European Congress; 2016; Vienna, Austria: Value in Health.

3. ZfKD, Robert Koch Institute. Krebsregisterdaten: German Centre for Cancer Registry Data 2016 [14th March 2017]. Available from: <www.krebsdaten.de>.

4. Rosenberg PS, Check DP, Anderson WF. A web tool for age-period-cohort analysis of cancer incidence and mortality rates. Cancer Epidemiol Biomarkers Prev. 2014;23(11):2296-302. doi: 10.1158/1055-9965.EPI-14-0300. PubMed PMID: 25146089; PubMed Central PMCID: PMCPMC4221491.

5. Holford TR, Cronin KA, Mariotto AB, Feuer EJ. Changing patterns in breast cancer incidence trends. J Natl Cancer Inst Monogr. 2006;(36):19-25. doi: 10.1093/jncimonographs/lgj016. PubMed PMID: 17032890.

6. Gangnon RE, Sprague BL, Stout NK, Alagoz O, Weedon-Fekjaer H, Holford TR, et al. The contribution of mammography screening to breast cancer incidence trends in the United States: an updated age-period-cohort model. Cancer Epidemiol Biomarkers Prev. 2015;24(6):905-12. doi: 10.1158/1055-9965.EPI-14-1286. PubMed PMID: 25787716; PubMed Central PMCID: PMCPMC4489135.

7. Berkemeyer S, Lemke D, Hense HW. Incidence and Mortality Trends in German Women with Breast Cancer Using Age, Period and Cohort 1999 to 2008. PLoS One. 2016;11(3):e0150723. doi: 10.1371/journal.pone.0150723. PubMed PMID: 26933878; PubMed Central PMCID: PMCPMC4774986.

8. Federal Statistical Office. GENESIS-Online. Wiesbaden: Statistisches Bundesamt (Destatis); 2017.

9. Kaatsch P, Spix C, Hentschel S, Katalinic A. Krebs in Deutschland 2009/2010. 2013. doi: papers2://publication/uuid/2DBC4F9F-5A6E-4B21-BAA0-5BD8042ECFF0.

10. Hemminki K, Bermejo JL. Effects of screening for breast cancer on its age-incidence relationships and familial risk. Int J Cancer. 2005;117(1):145-9. doi: 10.1002/ijc.21149. PubMed PMID: 15880571.

11. Trentham-Dietz A, Kerlikowske K, Stout NK, Miglioretti DL, Schechter CB, Ergun MA, et al. Tailoring Breast Cancer Screening Intervals by Breast Density and Risk for Women Aged 50 Years or Older: Collaborative Modeling of Screening Outcomes. Ann Intern Med. 2016;165(10):700-12. doi: 10.7326/M16-0476. PubMed PMID: 27548583; PubMed Central PMCID: PMCPMC5125086.

12. Vilaprinyo E, Forné C, Carles M, Sala M, Pla R, Castells X, et al. Cost-Effectiveness and Harm-Benefit Analyses of Risk-Based Screening Strategies for Breast Cancer. PloS one. 2014;9(2):e86858.

13. Breast Cancer Surveillance Consortium. Breast Cancer Surveillance Consortium Risk Calculator Maryland: Breast Cancer Surveillance Consortium,

National Cancer Institute,; 2015 [20th March 2017]. Available from: <https://tools.bcsc-scc.org/bc5yearrisk/calculator.htm>.

14. BCSC. Breast Cancer Surveillance Consortium (BCSC). In: BCSC, editor. Risk Factors Dataset2016.

15. Work ME, Reimers LL, Quante AS, Crew KD, Whiffen A, Terry MB. Changes in mammographic density over time in breast cancer cases and women at high risk for breast cancer. Int J Cancer. 2014;135(7):1740-4. doi: 10.1002/ijc.28825. PubMed PMID: 24599445; PubMed Central PMCID: PMCPMC4107003.

16. Kerlikowske K, Ichikawa L, Miglioretti DL, Buist DSM, Vacek PM, Smith-Bindman R, et al. Longitudinal Measurement of Clinical Mammographic Breast Density to Improve Estimation of Breast Cancer Risk. JNCI Journal of the National Cancer Institute. 2007;99(5):386-95. doi: papers2://publication/doi/10.1093/jnci/djk066.

17. Weigel S, Heindel W, Heidrich J, Hense HW, Heidinger O. Digital mammography screening: sensitivity of the programme dependent on breast density. Eur Radiol. 2016. doi: 10.1007/s00330-016-4636-4. PubMed PMID: 27822617.

18. Tice JA, Cummings SR, Smith-Bindman R, Ichikawa L, Barlow WE, Kerlikowske K. Using clinical factors and mammographic breast density to estimate breast cancer risk: development and validation of a new predictive model. Annals of internal medicine. 2008;148(5):337-47.

19. Boyd NF, Guo H, Martin LJ, Sun L, Stone J, Fishell E, et al. Mammographic density and the risk and detection of breast cancer. N Engl J Med. 2007;356(3):227-36. doi: 10.1056/NEJMoa062790. PubMed PMID: 17229950.

20. Kerlikowske K, Zhu W, Hubbard RA, Geller B, Dittus K, Braithwaite D, et al. Outcomes of screening mammography by frequency, breast density, and postmenopausal hormone therapy. JAMA Intern Med. 2013;173(9):807-16. doi: 10.1001/jamainternmed.2013.307. PubMed PMID: 23552817; PubMed Central PMCID: PMCPMC3699693.

21. Biesheuvel C, Weigel S, Heindel W. Mammography Screening: Evidence, History and Current Practice in Germany and Other European Countries. Breast Care (Basel). 2011;6(2):104-9. doi: 10.1159/000327493. PubMed PMID: 21673820; PubMed Central PMCID: PMCPMC3104900.

22. Deutsches Mammographie-Screening-Programm. Jahresbericht Evaluation 2014. Berlin: Kooperationsgemeinschaft Mammographie, 2016.

23. Kooperationsgemeinschaft Mammographie. Jahresbericht Qualitätssicherung 2014. Ergebnisse des Mammographie-Screening-Programms in Deutschland. Berlin: Kooperationsgemeinschaft Mammographie,, 2016.

24. Tumorregister München. ICD-10 C50: Mammakarzinom (Frauen) - Survival. München: Tumorregister München, 2016.

25. Young JJ, Roffers S, Ries L, Fritz A. SEER Summary Staging Manual - 2000: Codes and Coding Instructions. Bethesda, MD: National Cancer Institute, 2001.

26. Arndt V, Sturmer T, Stegmaier C, Ziegler H, Dhom G, Brenner H. Socio-demographic factors, health behavior and late-stage diagnosis of breast cancer in Germany: a population-based study. J Clin Epidemiol. 2001;54(7):719-27. PubMed PMID: 11438413.

27. Rosenberg MA. Competing risks to breast cancer mortality. J Natl Cancer Inst Monogr. 2006;(36):15-9. doi: 10.1093/jncimonographs/lgj004. PubMed PMID: 17032889.

28. Mandelblatt J, Cronin K, de Koning H, Miglioretti D, Schechter C, Stout N. Collaborative Modeling of U.S. Breast Cancer Screening Strategies. Writing Committee of the Breast Cancer Working Group Cancer Intervention and Surveillance Modeling Network (CISNET) and the Breast Cancer Surveillance Consortium (BCSC), 2015.

29. Kassenärztliche Bundesvereinigung. Einheitlicher Bewertungsmaßstab für ärztliche Leistungen Berlin: Kassenärztliche Bundesvereinigung,; 2017 [24th March 2017]. Available from: <http://www.kbv.de/html/index.php>.

30. Liberman L, Dershaw DD, Rosen PP, Abramson AF, Deutch BM, Hann LE. Stereotaxic 14-gauge breast biopsy: how many core biopsy specimens are needed? Radiology. 1994;192(3):793-5. doi: 10.1148/radiology.192.3.8058949. PubMed PMID: 8058949.

31. Apesteguia L, Pina LJ. Ultrasound-guided core-needle biopsy of breast lesions. Insights Imaging. 2011;2(4):493-500. doi: 10.1007/s13244-011-0090-7. PubMed PMID: 22347970; PubMed Central PMCID: PMCPMC3259303.

32. Muller D, Danner M, Rhiem K, Stollenwerk B, Engel C, Rasche L, et al. Cost-effectiveness of different strategies to prevent breast and ovarian cancer in German women with a BRCA 1 or 2 mutation. Eur J Health Econ. 2017. doi: 10.1007/s10198-017-0887-5. PubMed PMID: 28382503.

33. German Consortium for Hereditary Breast and Ovarian Cancer. Zentren Für Familiären Brust- und Eierstockkrebs Bonn: Stiftung Deutsche Krebshilfe; 2017 [10th April 2017]. Available from: <https://www.krebshilfe.de/helfen/rat-hilfe/familiaerer-krebs/zentren-fuer-familiaeren-brust-und-eierstockkrebs/>.

34. Liedtke C, Rody A, Gluz O, Baumann K, Beyer D, Kohls EB, et al. The prognostic impact of age in different molecular subtypes of breast cancer. Breast Cancer Res Treat. 2015;152(3):667-73. doi: 10.1007/s10549-015-3491-3. PubMed PMID: 26195120.

35. Kreienberg R, Albert U-S, Follmann M, Kühn T, Wöckel A, Zemmler T. Interdisziplinäre S3-Leitlinie für die Diagnostik, Therapie und Nachsorge des Mammakarzinoms - Langversion. Leitlinienprogramm Onkologie, 2012.

36. Engel J, Nagel G, Breuer E, Meisner C, Albert US, Strelocke K, et al. Primary breast cancer therapy in six regions of Germany. Eur J Cancer. 2002;38(4):578-85. PubMed PMID: 11872353.

37. DeKoven M, Bonthapally V, Jiao X, Ganguli A, Pathak P, Lee WC, et al. Treatment pattern by hormone receptors and HER2 status in patients with metastatic breast cancer in the UK, Germany, France, Spain and Italy (EU-5): results from a physician survey. J Comp Eff Res. 2012;1(5):453-63. doi: 10.2217/cer.12.43. PubMed PMID: 24236422.

38. KVRLP. GE-Bericht DMP Brustkrebs 01-06/2016 Mainz: Kassenärztliche Vereinigung Rheinland-Pfalz; 2016 [6th June, 2017]. Available from: <https://www.kv-rlp.de/mitglieder/dmp/dmp-brustkrebs/>.

39. Lux MP, Wockel A, Benedict A, Buchholz S, Kreif N, Harbeck N, et al. Cost-effectiveness analysis of anastrozole versus tamoxifen in adjuvant therapy for early-stage breast cancer - a health-economic analysis based on the 100-month analysis of the ATAC trial and the German health system. Onkologie. 2010;33(4):155-66. doi: 10.1159/000286233. PubMed PMID: 20389141.

40. WIdO. Arzneiverordnungs-Report Bonn: Wissenschaftliches Institut der AOK; 2017 [cited 6th June, 2017]. Available from: <https://www.wido.de/arz_themenueberblick.html>.

41. Schwabe U, Paffrath D. Arzneimittelverordnungsreport. Berlin: Springer-Verlag Berlin Heidelberg; 2014.

42. Lidgren M, Wilking N, Jönsson B, Rehnberg C. Health related quality of life in different states of breast cancer. Qual Life Res. 2007;16(6):1073-81. doi: papers2://publication/doi/10.1007/s11136-007-9202-8.

43. Dolan P. Modeling valuations for EuroQol health states. Med Care. 1997;35(11):1095-108. PubMed PMID: 9366889.

44. Burström K, Sun S, Gerdtham U-G, Henriksson M, Johannesson M, Levin L-Å, et al. Swedish experience-based value sets for EQ-5D health states. Quality of Life Research. 2014;23(2):431-42.

45. de Haes JC, de Koning HJ, van Oortmarssen GJ, van Agt HM, de Bruyn AE, van Der Maas PJ. The impact of a breast cancer screening programme on quality-adjusted life-years. Int J Cancer. 1991;49(4):538-44. PubMed PMID: 1917155.

46. Bonomi AE, Boudreau DM, Fishman PA, Ludman E, Mohelnitzky A, Cannon EA, et al. Quality of life valuations of mammography screening. Qual Life Res. 2008;17(5):801-14. doi: papers3://publication/doi/10.1007/s11136-008-9353-2.

47. Tosteson AN, Fryback DG, Hammond CS, Hanna LG, Grove MR, Brown M, et al. Consequences of false-positive screening mammograms. JAMA Intern Med. 2014;174(6):954-61. doi: 10.1001/jamainternmed.2014.981. PubMed PMID: 24756610; PubMed Central PMCID: PMCPMC4071565.

48. Verkooijen HM, Buskens E, Peeters PH, Borel Rinkes IH, de Koning HJ, van Vroonhoven TJ, et al. Diagnosing non-palpable breast disease: short-term impact on quality of life of large-core needle biopsy versus open breast biopsy. Surg Oncol. 2002;10(4):177-81. PubMed PMID: 12020672.

49. Domeyer PJ, Sergentanis TN, Zagouri F, Zografos GC. Health-related quality of life in vacuum-assisted breast biopsy: short-term effects, long-term effects and predictors. Health Qual Life Outcomes. 2010;8:11. doi: 10.1186/1477-7525-8-11. PubMed PMID: 20102642; PubMed Central PMCID: PMCPMC2835677.

50. Tagliafico A, Gristina L, Bignotti B, Valdora F, Tosto S, Calabrese M. Effects on short-term quality of life of vacuum-assisted breast biopsy: comparison between digital breast tomosynthesis and digital mammography. Br J Radiol. 2015;88(1056):20150593. doi: 10.1259/bjr.20150593. PubMed PMID: 26463101; PubMed Central PMCID: PMCPMC4984943.

51. Bundred SM, Maxwell AJ, Morris J, Lim YY, Harake MJ, Whiteside S, et al. Randomized controlled trial of stereotactic 11-G vacuum-assisted core biopsy for the diagnosis and management of mammographic microcalcification. Br J Radiol. 2016;89(1058):20150504. doi: 10.1259/bjr.20150504. PubMed PMID: 26654214; PubMed Central PMCID: PMCPMC4985199.

52. Maxwell JR, Bugbee ME, Wellisch D, Shalmon A, Sayre J, Bassett LW. Imaging-Guided Core Needle Biopsy of the Breast: Study of Psychological Outcomes. Breast J. 2000;6(1):53-61. PubMed PMID: 11348335.

53. Zagouri F, Sergentanis TN, Gounaris A, Koulocheri D, Nonni A, Domeyer P, et al. Pain in different methods of breast biopsy: emphasis on vacuum-assisted breast biopsy. Breast. 2008;17(1):71-5. doi: 10.1016/j.breast.2007.07.039. PubMed PMID: 17869106.

54. Hemmer JM, Kelder JC, van Heesewijk HP. Stereotactic large-core needle breast biopsy: analysis of pain and discomfort related to the biopsy procedure. Eur Radiol. 2008;18(2):351-4. doi: 10.1007/s00330-007-0762-3. PubMed PMID: 17909818; PubMed Central PMCID: PMCPMC2668619.
